# Area-Driven Adaptive Sampling of Closed Droplet Contours for Vision-Based Droplet Observation

**DOI:** 10.3390/jimaging12090402

**Published:** 2026-08-26

**Authors:** Xuefeng Wang, Yangting Zheng, Chenyao Bai, Yinqi Chen, Xiang Gao, Yiyue Li, Yunlong Zhu

**Affiliations:** 1 College of Intelligent Robotics and Advanced Manufacturing, Fudan University, Shanghai 200433, China; 22110860040@m.fudan.edu.cn (X.W.); ytzheng24@m.fudan.edu.cn (Y.Z.); 22110860032@m.fudan.edu.cn (Y.L.); zyl@fudan.edu.cn (Y.Z.); 2 Jihua Laboratory, Foshan 528200, China; chenyq277@alumni.sysu.edu.cn (Y.C.); gaoxiang@jihualab.com (X.G.)

**Keywords:** closed-contour sampling, droplet metrology, area-driven sampling, area estimation, sampling-budget estimation, vision-based droplet observation

## Abstract

Closed droplet contours provide the geometric basis for area estimation in vision-based droplet observation. In OLED inkjet printing, droplets are deposited into pixel wells with predefined geometry; projected area is therefore a primary geometric quantity for assessing whether the deposited liquid sufficiently fills the well or risks overflow. This work formulates closed-contour sampling under a fixed sampling budget as an area-driven sampling problem. A leading-order analysis of the local arc–chord area error shows that the dominant cubic term depends jointly on curvature and segment length. Minimization of the resulting leading-order area-error functional yields an asymptotically optimal area-driven sampling density proportional to the cube root of curvature, together with a sampling-budget estimate under a target area-error tolerance. The derived sampling density is implemented on the fitted closed contour through cumulative-weight inversion. Experiments on random closed curves and the droplet dataset provide a systematic quantitative comparison with representative methods under identical fixed-budget settings, complemented by statistical analysis and evaluations of geometric fidelity, sensitivity, and computational efficiency. The proposed method achieves lower area estimation error under the tested sampling budgets, with the improvement being most pronounced at lower sampling budgets, while the reported geometric-fidelity metrics show no disproportionate degradation of contour fidelity. These results demonstrate the effectiveness of area-driven sampling for closed-contour area estimation under limited sampling budgets.

## 1. Introduction

**Figure 1:**
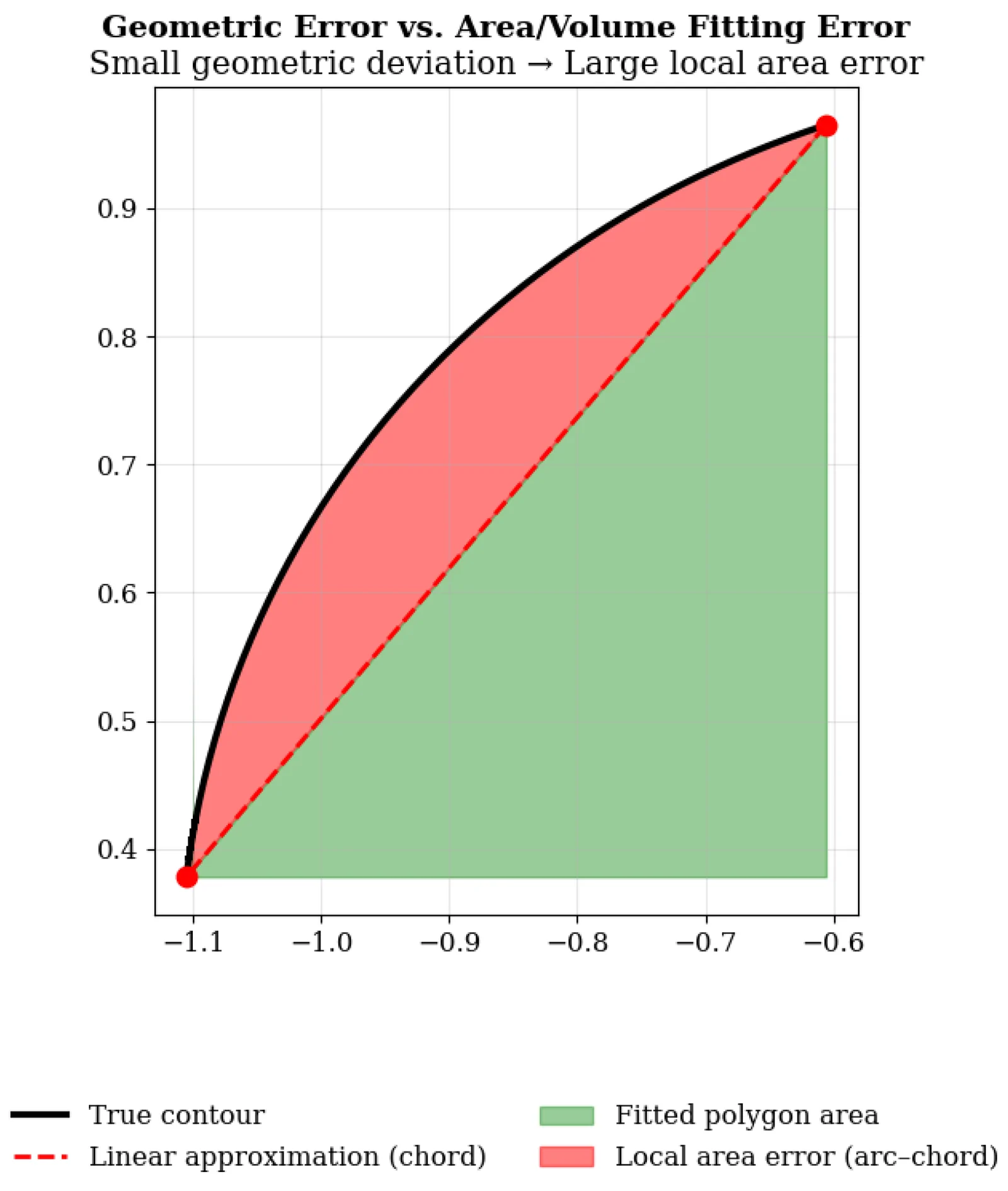
Approximation error introduced by discretizing the contour.

In vision-based droplet observation, images serve as the geometric foundation for a range of downstream tasks, including projected-area estimation, volume inversion, morphology-stability analysis, and process-parameter optimization. Among all observable features, the closed contour extracted from droplet images provides the most direct and stable link between the image domain and physically meaningful quantities [1,2,3], such as projected area, equivalent diameter, roundness, and eccentricity. These quantities hold direct engineering importance in applications like inkjet printing, liquid dispensing, and spray systems [4,5,6,7]. Nevertheless, for a closed contour with an arbitrary shape, its enclosed area is ultimately evaluated after discretizing the contour into an ordered set of boundary points, which are represented by a polygonal approximation. As a result, the area computed from a discretely sampled polygon inevitably incurs approximation errors (Figure 1). This observation leads directly to the central problem addressed in this paper: given a closed droplet contour, how should it be sampled to maximize the fidelity of the estimated area?

In OLED inkjet printing, droplets are deposited into pixel wells with predefined geometry, and the projected droplet area directly reflects the filling state of the well. Insufficient projected area may correspond to incomplete filling, whereas excessive area increases the risk of overflow. Accurate area estimation from a limited number of contour samples is therefore of direct practical relevance to droplet observation in this process, providing the application basis for the area-driven sampling formulation developed in this study. Although droplet observation provides the primary application context, the formulation itself is defined on a sufficiently smooth fitted planar closed contour and does not depend on a droplet-specific shape model. Random closed curves are therefore included to evaluate the sampling law beyond the particular geometries observed in the droplet dataset. A limited contour-point budget is particularly relevant to high-frame-rate observation and resource-constrained contour representations, where the retained boundary points directly affect storage, transmission, and subsequent computational cost.

Classical studies on contour discretization have investigated contour simplification, curve reconstruction, and adaptive sampling or representation. Contour-simplification methods reduce the number of contour points under geometric approximation criteria [8,9,10,11,12,13,14], curve-reconstruction methods recover continuous curves from sparse, unordered, or noisy samples [15,16,17,18], and adaptive sampling methods allocate samples or control points according to local geometric information to improve approximation fidelity and representation efficiency [19,20,21,22,23]. More recent studies have further extended adaptive contour representation through parametric, learning-based, and hierarchical formulations [24,25,26,27,28,29,30,31].

Among these directions, contour simplification and adaptive sampling are the most directly comparable to the present problem in terms of their input–output form: they take a contour representation as input and produce a finite set of representative points or a polygonal approximation. The main distinction lies in the optimization objective. Existing methods primarily allocate or retain points to control geometric quantities such as point-to-curve deviation, chord error, curvature representation, or overall reconstruction fidelity, which is appropriate when the sampled contour is intended to preserve geometric shape. In the droplet-observation problem considered here, the retained contour points are subsequently used to estimate the enclosed projected area. The present method therefore retains the same general contour-to-finite-point representation framework but derives the sampling distribution directly from the area error introduced by polygonal discretization.

Nevertheless, these studies primarily optimize quantities such as reconstruction error, control-point efficiency, geometric smoothness, or visual fidelity. They do not directly address the error in the area estimation induced by contour discretization. This distinction is particularly important for droplet observation, as a minimum reconstruction error does not necessarily imply a minimum area error.

For area estimation, contour-based droplet measurement has repeatedly emphasized the close relationship between extracted boundaries and projected-area or volume computation [4,5,6,7]. Even so, most approaches treat sampling as a mere implementation step following contour extraction rather than as an independent optimization objective. In other words, the literature has largely focused on improving the reconstruction quality of closed contours, but has far less frequently addressed the design of contour sampling mechanisms specifically aimed at more accurate area estimation.

Area preservation has also been investigated in parametric and polygonal approximation [32,33,34,35]. These approaches preserve area through geometric operations such as parametric transformation, vertex relocation, edge movement, segment collapse, or the introduction of new vertices. In the present problem, however, the fitted contour itself is fixed and only the locations of a prescribed number of samples on that contour are allowed to vary. The geometric operations on which these area-preserving methods rely are therefore not admissible under the fixed-contour sampling constraint; directly restricting their output vertices to the fitted contour would change their original optimization mechanism rather than provide an equivalent sampling formulation.

Motivated by these observations, this paper reformulates the processing of arbitrary closed droplet contours as an area-driven sampling problem. The objective is neither to produce a visually smoother curve, nor to achieve a more compact representation, nor to obtain a curve closer to the original contour; instead, under a fixed sampling budget, it aims to maximize the accuracy of area estimation from the contour sampling. The proposed method is formulated directly with respect to area estimation error. Starting from the leading-order local arc–chord area error, the sampling density is derived by minimizing the corresponding leading-order error functional; this optimality is interpreted asymptotically. The same global area-error model yields a leading-order estimate of the sampling budget associated with a prescribed area-error tolerance. The experiments compare the proposed method with representative sampling methods under identical sampling budgets and report area estimation accuracy together with geometric-fidelity metrics used to assess possible geometric degradation.

## 2. Related Work

The related literature can be organized into five categories: contour simplification, curve reconstruction, adaptive sampling and contour representation, contour-based droplet measurement, and area-preserving approximation.

(1) **Contour simplification** aims to reduce the number of contour points while preserving the geometric shape of the boundary as much as possible. The early work of [8,9] established the basic paradigm of recursively splitting the curve at the point of maximum perpendicular distance. Ref. [10] detected dominant points by combining curvature and cornerity measures with a region of support. Ref. [11] introduced a simplification criterion that iteratively eliminates points with the smallest contribution. Ref. [12] developed a dynamic algorithm that minimizes the maximum approximation error for a fixed number of segments. Ref. [13] extended optimal polygonal approximation to closed discrete curves by handling cyclic boundary conditions. Ref. [14] proposed a greedy approximation strategy that progressively removes redundant points while stabilizing the remaining vertices.
(2) **Curve reconstruction** aims to recover continuous and plausible curves from extreme conditions [15]. One representative work reconstructs curves from unorganized points by combining moving least squares with Euclidean minimum spanning tree refinement [16]. To handle noisy samples, another method exploits crust and medial axis filtering [17]. Sampling sufficiency for reliable reconstruction has also been systematically analyzed [18].
(3) **Adaptive sampling and contour representation** seeks to improve geometric fidelity and representation efficiency under limited samples or control points. In adaptive sampling, ref. [19] pointed out the inefficiency of uniform sampling under high accuracy requirements, and ref. [20] further systematized curvature-based sampling for curves and surfaces. More recently, refs. [21,22] proposed near-optimal strategies based on deviation analysis and sectional error equivalence, respectively, while ref. [23] introduced an adaptive strategy based on maximum corresponding point deviation. Parametric and learned contour representations include persistent-homology-based reconstruction [24], learning-based control point prediction [25], neural-network-based knot solving for B-spline approximation [26], contour reconstruction based on concavity and convexity [27], perceptually faithful reconstruction by combining persistent homology with principal curves [28], image-processing-driven modeling and reconstruction via dual-channel detection and B-spline analysis [29], hierarchical local contour representation in AdaContour [30], and learning-based vector simplification with geometric-property evaluation [31].
(4) **Contour-based droplet measurement** uses image-extracted droplet boundaries to obtain projected area, equivalent geometric descriptors, and volume-related quantities for droplet observation and process analysis [4,5,6,7].
(5) **Area-preserving approximation** has been investigated in parametric and polygonal representations. Area-preserving transformations have been considered in NURBS and CAD [32]. Bose et al. studied area-preserving approximations of polygonal paths [33], Buchin et al. developed area-preserving simplification and schematization through edge-move operations [34], and Kronenfeld et al. proposed an area-preserving segment-collapse strategy [35].

Overall, the most directly related contour-simplification and adaptive-sampling methods are primarily designed to preserve geometric fidelity or representation efficiency, whereas contour-based droplet observation ultimately uses the retained boundary samples to estimate projected area. This difference in downstream objective makes sampling-induced area error a more directly aligned criterion for the droplet-observation setting considered here, motivating the area-driven sampling formulation developed in the following section.

## 3. Methods

### 3.1. Contour Extraction and Closed-Curve Representation

The proposed method takes an ordered closed contour obtained after image processing as input. In the droplet dataset experiments, image acquisition, preprocessing, binary segmentation, and dense contour extraction precede the sampling stage, which starts from the resulting ordered contour. After duplicate points are removed and the contour orientation is normalized, the contour is represented by a periodic cubic spline. For a parametric contour,

(1) r(t)=(x(t),y(t)),t∈[0,T],

subject to

(2) r(0)=r(T).

The ordered contour is represented by a periodic cubic spline, resulting in a $C^{2}$ closed curve with well-defined tangent, arc length, and curvature [36,37]. This smooth representation provides the geometric quantities required by the subsequent area-error analysis and sampling-density construction. Since curvature is determined by first- and second-order derivatives of the fitted curve, its numerical estimate naturally depends on the contour representation and spline fitting. The same fitted spline is used as the common contour representation for all sampling methods in the experiments.

### 3.2. Problem Formulation for Droplet Area Estimation

Let $\Omega \subset R^{2}$ denote the region enclosed by the fitted closed contour, and let its counterclockwise boundary be parameterized by arc length as

(3) r(s)=(x(s),y(s)),s∈[0,L],

where *L* is the total arc length. Its enclosed reference area is denoted by

(4) $$A={\;\int \int \;}_{\Omega }dA.$$

For a prescribed sampling budget of *N* distinct points on a closed curve, the sampling parameters are defined by

(5) $$0\leq s_{1}<s_{2}<\cdots <s_{N}<L,\;s_{N+1}=s_{1}+L.$$

The corresponding contour points are

(6) $$P_{i}=r\left(s_{i}\right),\;i=1,2,\ldots ,N.$$

Connecting the points in cyclic order yields a sampling polygon $P_{N}$, whose area is computed by the shoelace formula [38],

(7) $$A_{N}=\frac{1}{2}\left|\sum_{i=1}^{N}\left(x_{i}y_{i+1}-x_{i+1}y_{i}\right)\right|,\;P_{N+1}=P_{1}.$$

The area-estimation error used in the main experiments is

(8) $$E_{N}=\left|A-A_{N}\right|.$$

**Figure 2:**
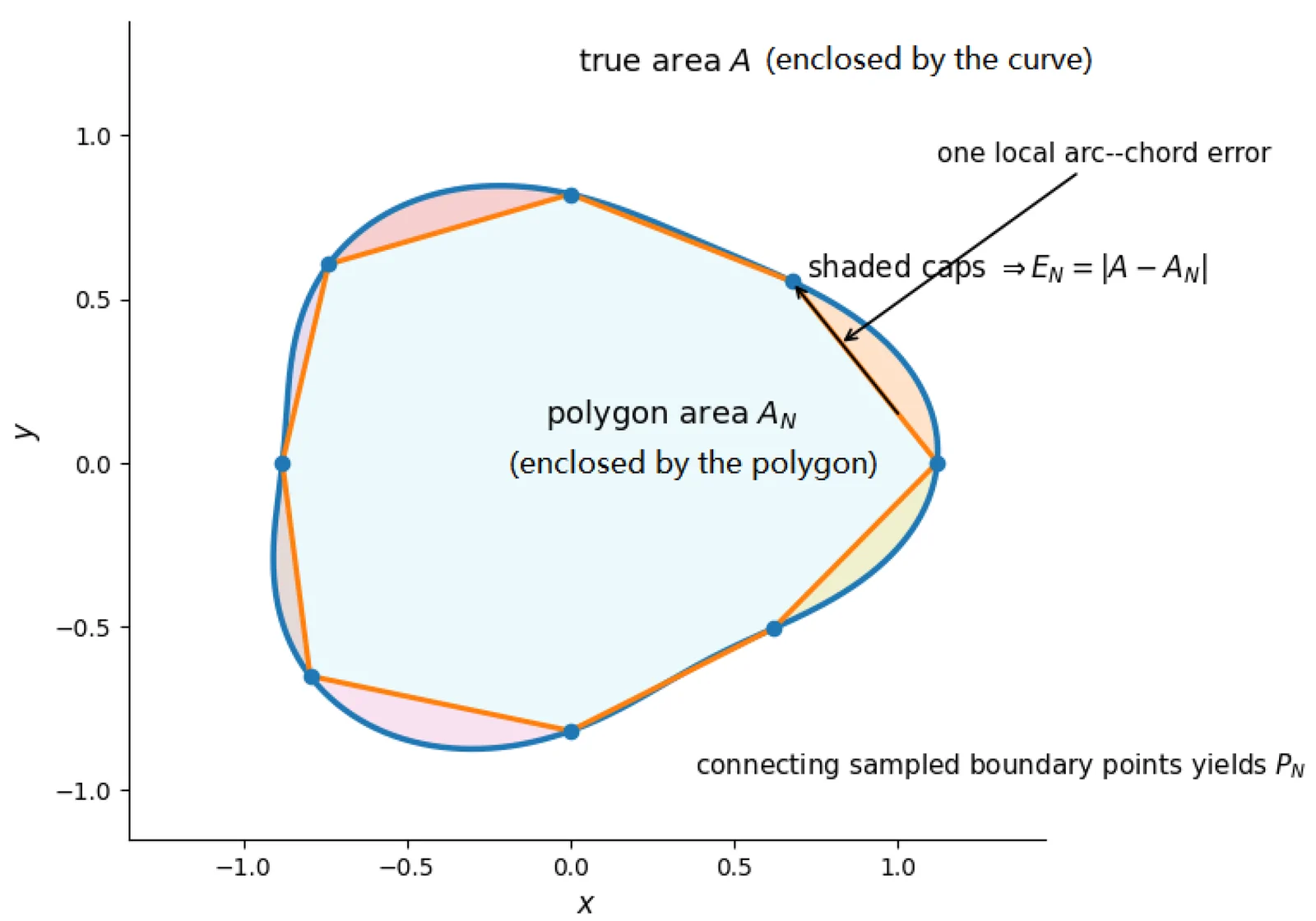
Illustration of the polygonal area-estimation error.

Accordingly, $E_{N}$ quantifies the sampling-induced geometric area error between the fitted reference contour and its polygonal approximation $P_{N}$ (Figure 2). The droplet dataset results use this fitted contour as the reference for evaluating the contribution of the contour-sampling stage, while acquisition, segmentation, localization, optical, calibration, and physical-reference uncertainties belong to the broader measurement pipeline.

### 3.3. Global Arc–Chord Area Error Model

Let the arc between adjacent samples $P_{i}$ and $P_{i+1}$ be denoted by $\Gamma_{i}$. Its arc length is

(9) $$\ell_{i}=s_{i+1}-s_{i},$$

and the corresponding chord length is

(10) $$c_{i}={∥P_{i+1}-P_{i}∥}_{2}.$$

Let $\Delta A_{i}$ be the signed area enclosed by $\Gamma_{i}$ and its chord. For sufficiently fine sampling, this local arc–chord discrepancy is governed, to leading order, by the local curvature level and the segment length. This relation provides the local area-error model used in the subsequent density formulation.

**Lemma** **1** (Local leading-order arc–chord area estimate)**.** *For a sufficiently short arc segment $\Gamma_{i}$, the corresponding arc–chord area error satisfies the leading-order estimate*

(11) $$|\Delta A_{i}|\;≲\frac{\kappa_{i}^{\max}\;\ell_{i}^{3}}{12},$$

*where $\kappa_{i}^{\max}$ is the maximum curvature on $\Gamma_{i}$. Here, *≲* denotes the leading-order short-arc estimate used in the subsequent continuum model rather than a universal finite-arc upper bound.*

**Figure 3:**
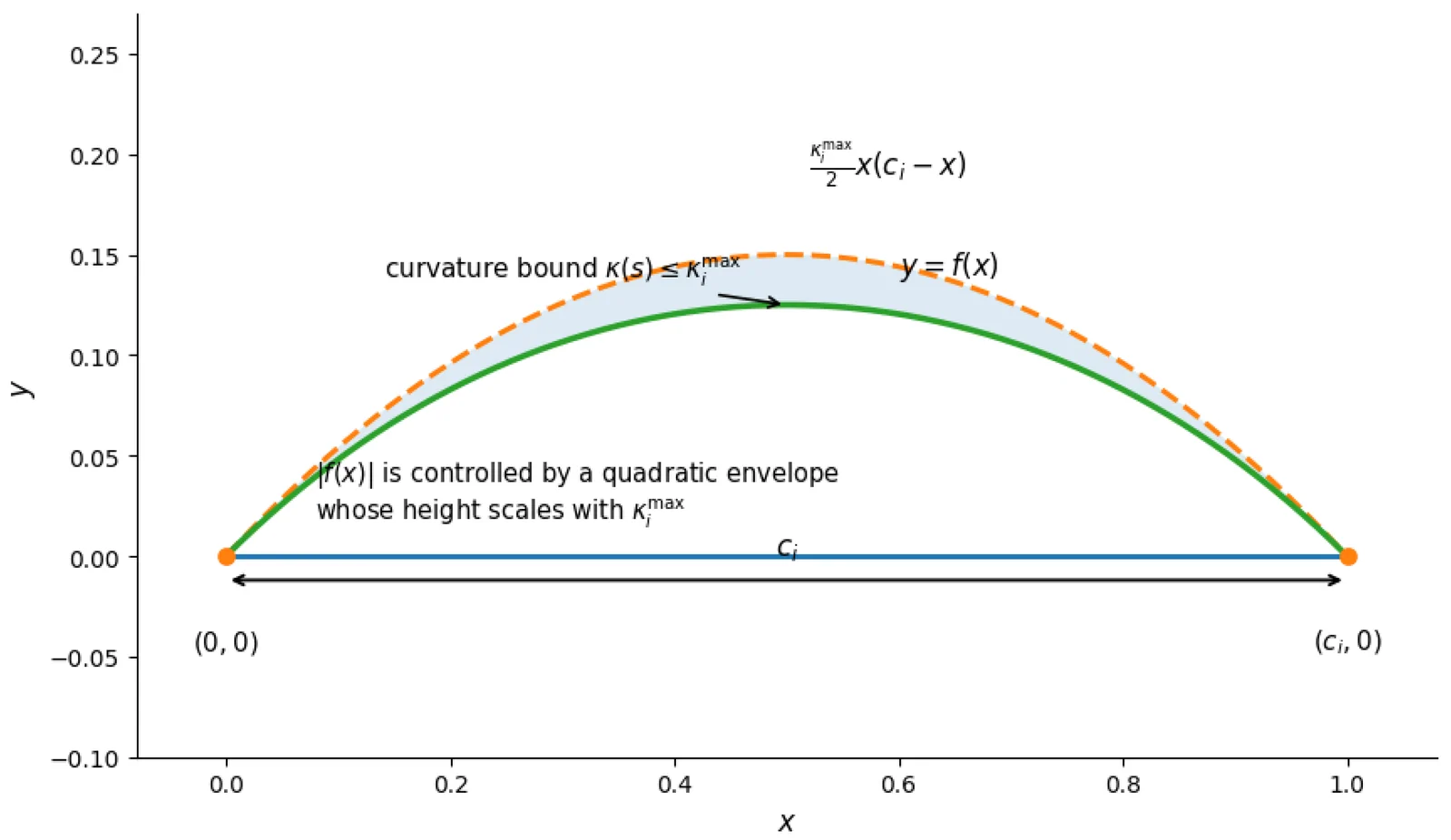
Geometric illustration of the local arc–chord area discrepancy on an arc segment $\Gamma_{i}$. The true arc y=f(x) is represented by a short circular arc, while the dashed curve illustrates the quadratic profile $\frac{\kappa_{i}^{\max}}{2}x\left(c_{i}-x\right)$ used in the local second-order approximation.

**Proof.**  In a local coordinate system, place the chord of $\Gamma_{i}$ on the *x*-axis with endpoints (0,0) and $\left(c_{i},0\right)$. The arc can then be written locally as y=f(x) for $x\in [0,c_{i}]$, with $f\left(0\right)=f\left(c_{i}\right)=0$. Because the contour is $C^{2}$ and the arc segment is sufficiently short, the deviation of the arc from its chord admits a second-order description governed by the local curvature scale (Figure 3). This follows from the local Taylor expansion of a smooth planar curve together with the standard relation between second-order geometry and curvature for arc-length-localized representations [36,39]. The corresponding curvature-controlled quadratic profile therefore gives the leading-order estimate

(12) $$\left|f\left(x\right)\right|≲\frac{\kappa_{i}^{\max}}{2}x\left(c_{i}-x\right),\;x\in \left[0,c_{i}\right].$$

Integrating over the chord-aligned coordinate yields

(13) $$|\Delta A_{i}|\;=\left|\int_{0}^{c_{i}}f\left(x\right)\;dx\right|≲\frac{\kappa_{i}^{\max}\;c_{i}^{3}}{12}.$$

Since $c_{i}\leq \ell_{i}$, the same cubic-order estimate can be written as

(14) $$|\Delta A_{i}|\;≲\frac{\kappa_{i}^{\max}\;\ell_{i}^{3}}{12}.$$

For sufficiently short arcs, replacing chord length by arc length affects only higher-order terms and does not change the leading cubic-order relation used in the subsequent optimization. This gives (11).    □

**Theorem** **1** (Leading-order global arc–chord area-error model)**.** *Based on the definition of $E_{N}$ in *(8)* and the local estimate in Lemma 1, the accumulated area-error model is*

(15) $$E_{N}\leq \sum_{i=1}^{N}\left|\Delta A_{i}\right|≲\frac{1}{12}\sum_{i=1}^{N}\kappa_{i}^{\max}\;\ell_{i}^{3}.$$

**Proof.**  By the triangle inequality,

(16) $$E_{N}\leq \sum_{i=1}^{N}\left|\Delta A_{i}\right|.$$

Applying the leading-order estimate (11) to the *N* arc segments and summing gives the second relation in (15).    □

Theorem 1 makes explicit that, for a fixed sampling budget, the leading area-error contribution is governed jointly by the segment-length distribution $\left\{\ell_{i}\right\}$ and the local curvature distribution. As the sampling intervals become sufficiently short, $\kappa_{i}^{\max}$ represents the local curvature level on each shrinking arc segment, which provides the connection to the continuous curvature field κ(s) used in the density formulation. Equation (15) therefore serves as the analytical bridge from the discrete polygonal approximation problem to the leading-order continuum area-error functional considered next. Accordingly, the optimality of the resulting sampling law is interpreted asymptotically in the dense-sampling regime.

### 3.4. Asymptotically Optimal Area-Driven Sampling Density

To derive the area-driven sampling rule, we replace the discrete allocation of sample points by a continuum density description along the contour. Let s∈[0,L] denote the arc-length parameter, and let κ(s)≥0 be the curvature magnitude. We introduce a sampling density ρ(s) along arc length, interpreted as the local number of segments per unit arc length [40]. The total sampling budget then satisfies

(17) $$\int_{0}^{L}\rho \left(s\right)\;ds=N.$$

Accordingly, the local average segment length is approximated by

(18) $$\ell \left(s\right)\approx \frac{1}{\rho (s)}.$$

This reciprocal relation follows from the interpretation of ρ(s) as the local number of sampling segments per unit arc length: when ρ varies slowly near *s*, each local segment occupies approximately 1/ρ(s) units of arc length.

Using the leading-order relation in (11), a continuum density description is introduced to derive the area-driven sampling density. The resulting error functional provides a continuum representation of the leading-order accumulation of the absolute local arc–chord area error. Under the density description above, the number of local sampling segments associated with a sufficiently small interval $I_{\Delta s}=\left[s,s+\Delta s\right]$ is approximated by

(19) $$n\left(I_{\Delta s}\right)\approx \int_{s}^{s+\Delta s}\rho \left(u\right)\;du\approx \rho \left(s\right)\Delta s,$$

Provided that ρ varies slowly over $I_{\Delta s}$. Meanwhile, the leading-order contribution of each local segment is approximated by

(20) $$|\Delta A_{i}|\;≲\frac{1}{12}\kappa \left(s\right)\ell {\left(s\right)}^{3}\approx \frac{1}{12}\kappa \left(s\right)\rho {\left(s\right)}^{-3}.$$

Let $\Delta E(I_{\Delta s})$ denote the leading-order accumulated absolute arc–chord area contribution associated with $I_{\Delta s}$. Multiplying the local single-segment contribution by the number of local segments gives

(21) $$\Delta E\left(I_{\Delta s}\right)≲n\left(I_{\Delta s}\right)\;\left|\Delta A_{i}\right|\approx \frac{1}{12}\kappa \left(s\right)\rho {\left(s\right)}^{-2}\Delta s.$$

Replacing the discrete summation by its continuum counterpart yields the total leading-order area-error functional E[ρ],

(22) $$E\left[\rho \right]=\frac{1}{12}\int_{0}^{L}\kappa \left(s\right)\rho {\left(s\right)}^{-2}\;ds.$$

Thus, the sampling-density problem is to minimize E[ρ] subject to (17). The resulting density minimizes the leading-order error functional and provides the asymptotic sampling law for the discrete area-error problem as the maximum segment length tends to zero.

**Theorem** **2** (Asymptotically optimal area-driven sampling density)**.** *Assume κ(s)≥0 on [0,L] and $\kappa^{1/3}\in L^{1}\left(\left[0,L\right]\right)$. Consider the admissible set*

$$A_{N}:=\left\{\rho :\left[0,L\right]\to \left[0,\infty \right)\;|\;\int_{0}^{L}\rho \left(s\right)\;ds=N\right\}.$$

*For the error functional*

(23) $$E\left[\rho \right]=\frac{1}{12}\int_{0}^{L}\kappa \left(s\right)\rho {\left(s\right)}^{-2}\;ds,$$

*the minimizer of this leading-order continuum functional over $A_{N}$ is*

(24) $$\rho^{★}\left(s\right)=\frac{N\;\kappa {\left(s\right)}^{1/3}}{\int_{0}^{L}\kappa {\left(u\right)}^{1/3}\;du},$$

*and the corresponding minimum value is*

(25) $$E_{\min}=\frac{1}{12N^{2}}{\left(\int_{0}^{L}\kappa {\left(s\right)}^{1/3}\;ds\right)}^{3}.$$

**Proof.**  We minimize the functional E[ρ] under the budget constraint $\int_{0}^{L}\rho \left(s\right)\;ds=N$. Introduce a Lagrange multiplier λ and define the augmented functional

(26) $$J\left[\rho \right]=\frac{1}{12}\int_{0}^{L}\kappa \left(s\right)\rho {\left(s\right)}^{-2}\;ds+\lambda \left(\int_{0}^{L}\rho \left(s\right)\;ds-N\right).$$

Since the integrand involves ρ(s) only and does not involve its derivative $\rho^{\prime }\left(s\right)$, the Euler–Lagrange condition reduces to a pointwise algebraic equation:

(27) $$\frac{\partial }{\partial \rho }\left(\frac{1}{12}\kappa \left(s\right)\rho^{-2}+\lambda \rho \right)=0.$$

Hence

(28) $$-\frac{1}{6}\kappa \left(s\right)\rho {\left(s\right)}^{-3}+\lambda =0,$$

which gives

(29) $$\rho {\left(s\right)}^{3}=\frac{\kappa (s)}{6\lambda }.$$

Therefore the minimizer of the leading-order continuum functional must satisfy

(30) $$\rho^{★}\left(s\right)=C\;\kappa {\left(s\right)}^{1/3},$$

where $C={\left(6\lambda \right)}^{-1/3}$ is a constant independent of *s*.

Using the constraint $\int_{0}^{L}\rho^{★}\left(s\right)\;ds=N$, we obtain

(31) $$C\int_{0}^{L}\kappa {\left(s\right)}^{1/3}\;ds=N,$$

so that

(32) $$C=\frac{N}{\int_{0}^{L}\kappa {\left(u\right)}^{1/3}\;du}.$$

Substituting this into (30) yields (24).

Finally, substituting (24) into (23) gives

(33) $$\begin{array}{cc} E_{\min} & \;\;=\frac{1}{12}\int_{0}^{L}\kappa \left(s\right){\left(\frac{N\kappa {\left(s\right)}^{1/3}}{\int_{0}^{L}\kappa {\left(u\right)}^{1/3}\;du}\right)}^{-2}ds \end{array}$$

(34) $$\begin{array}{c} \;\;\;\;\;\;\;\;\;\;\;\;\;\;\;=\frac{1}{12}\frac{{\left(\int_{0}^{L}\kappa {\left(u\right)}^{1/3}\;du\right)}^{2}}{N^{2}}\int_{0}^{L}\kappa {\left(s\right)}^{1/3}\;ds \end{array}$$

(35) $$\begin{array}{c} =\frac{1}{12N^{2}}{\left(\int_{0}^{L}\kappa {\left(s\right)}^{1/3}\;ds\right)}^{3}. \end{array}$$

This proves (25).    □

Equation (24) shows that the minimizer of the leading-order continuum functional can be restated in terms of the local spacing law

(36) $$\ell^{★}\left(s\right)\propto \kappa {\left(s\right)}^{-1/3},$$

which is referred to as the area-driven sampling law. Its optimality is with respect to the leading-order error functional; at finite sampling budgets, it is interpreted asymptotically rather than as a strict finite-sample optimum.

### 3.5. Sampling-Budget Estimation Under a Target Area-Error Tolerance Constraint

Let $\varepsilon_{\mathrm{abs}}$ denote a target absolute area-error tolerance. Because the sampling density follows from the leading-order global area-error model, the corresponding expression below is interpreted as a leading-order estimate of the sampling budget rather than as an exact finite-sample minimum or deterministic guarantee.

**Corollary** **1** (Sampling-budget estimate under a target area-error tolerance)**.** *For the area-driven density, the leading-order surrogate predicts*

(37) $$\hat{N}=\left\lceil \sqrt{\frac{1}{12\varepsilon_{\mathrm{abs}}}{\left(\int_{0}^{L}\kappa {\left(s\right)}^{1/3}\;ds\right)}^{3}}\right\rceil .$$

The sampling-budget estimate $\hat{N}$ is evaluated by sampling the contour with $\hat{N}$ points and computing the resulting polygonal area error. Because $\hat{N}$ follows from the leading-order area-error model, the satisfaction rate is an empirical measure of the accuracy of this estimate and is not required to equal one.

### 3.6. Area-Driven Sampling Law from Arc Length Space to Parameter Space

The derivation above is formulated in the arc-length domain. In practice, however, the contour is represented by the parametric form (1). Therefore, in this section, the derived density law is transported from arc length to the curve parameter.

For the parametric form r(t), the arc-length differential is

(38) $$ds=|r^{\prime }\left(t\right)|dt.$$

Its curvature is given by the standard planar-parametric formula from differential geometry and spline theory [36,37]:

(39) $$\kappa \left(t\right)=\frac{|x^{\prime }\left(t\right)y^{\prime \prime }\left(t\right)-y^{\prime }\left(t\right)x^{\prime \prime }\left(t\right)|}{{\left(x^{\prime }{\left(t\right)}^{2}+y^{\prime }{\left(t\right)}^{2}\right)}^{3/2}}.$$

Under (30), the corresponding probability density in *s* is proportional to $\kappa {\left(s\right)}^{1/3}$. Since $p_{t}\left(t\right)dt=p_{s}\left(s\right)ds$, the parameter-domain density must satisfy

(40) $$p_{t}\left(t\right)\propto \kappa {\left(t\right)}^{1/3}\left|r^{\prime }\left(t\right)\right|.$$

Accordingly, the parameter-domain weight used in the numerical implementation is

(41) $$w\left(t\right)={\left(\kappa (t)+\epsilon \right)}^{1/3}\left|r^{\prime }\left(t\right)\right|,$$

where $\epsilon =10^{-6}$ is adopted in all main experiments (based on Section 6.3), lying well within the stable range in which the sampling results are essentially insensitive to its exact value. The small positive constant ϵ regularizes the curvature weight in zero- or near-zero-curvature regions, avoiding flat intervals in the cumulative-weight function while retaining the curvature-driven allocation over the rest of the contour. Since ϵ is added directly to κ(t), it is defined on the same numerical scale as the computed curvature.

The normalized cumulative weight function is then

(42) $$F\left(t\right)=\frac{\int_{0}^{t}w\left(\tau \right)d\tau }{\int_{0}^{T}w\left(\tau \right)d\tau }.$$

Equation (42) reformulates the density-design problem as a quantile allocation problem, where equal increments in the cumulative weight yield samples that follow the desired area-driven distribution. Consequently, the theoretical density law originally derived in the arc-length domain is translated into an explicit numerical sampling rule in the parameter domain. In this sense, the parameter-domain algorithm serves as a numerical implementation of the area-driven density derived from the leading-order continuum functional under the change of variables from *s* to *t*.

Let $t_{0}$ be the starting parameter and let *N* be the total number of samples. After transforming the derived arc-length density into the parameter domain, the sampling problem becomes one of equal allocation in the normalized cumulative weight. Specifically, if F(t) denotes the cumulative distribution induced by the parameter-domain weight function, then the sample parameters ${\left\{t_{i}\right\}}_{i=1}^{N}$ should partition the accumulated weight into *N* equal parts, up to a cyclic shift determined by $t_{0}$.

### 3.7. Implementation of the Area-Driven Sampling Algorithm

The area-driven sampling law is obtained in the arc-length domain from the density (24) derived from the leading-order continuum functional. After transformation into the parameter domain, the sampling locations are determined by equally partitioning the cumulative weight induced by the parameter-domain weight function w(t) defined in (41). Thus, the following procedure constitutes a discrete realization of the derived sampling law.

Let the parameter interval [0,T] be discretized into *M* subintervals with nodes ${\left\{t_{j}\right\}}_{j=0}^{M}$. The cumulative weight is first approximated by numerical quadrature and then inverted by interpolation to recover the target sample locations. The weights $w_{j}=w\left(t_{j}\right)$ and the cumulative integral are computed via the trapezoidal rule:

(43) $$W_{0}=0,\;W_{j}=W_{j-1}+\frac{w_{j-1}+w_{j}}{2}\left(t_{j}-t_{j-1}\right),\;j=1,2,\ldots ,M.$$

Here *M* denotes the number of auxiliary grid intervals used for numerical quadrature and inverse interpolation, while *N* is the final number of sampling points. A fixed ratio M/N=100 is used in all experiments, making the auxiliary grid two orders of magnitude finer than the target sampling scale. This conservative fixed resolution is used to reduce numerical quadrature and inverse-interpolation error while keeping the numerical realization identical across contours and sampling budgets.

Let $W_{\mathrm{tot}}=W_{M}$. On the auxiliary grid, the normalized cumulative function in (42) is represented by $F\left(t_{j}\right)=W_{j}/W_{\mathrm{tot}}$, so inversion of equal quantiles of *F* is equivalent to inversion of the cumulative weights $W_{j}$. Let $W(t_{0})$ denote the cumulative weight at the chosen starting point, obtained from the discretized cumulative integral. Define

(44) $$\Delta W=\frac{W_{\mathrm{tot}}}{N}.$$

The target cumulative values are then chosen as

(45) $$U_{i}=W\left(t_{0}\right)+\left(i-1\right)\;\Delta W,\;i=1,2,\ldots ,N,$$

which is the discrete realization of equal partition in cumulative weight starting from the prescribed initial parameter $t_{0}$. Whenever $U_{i}\geq W_{\mathrm{tot}}$, a wrapping operation is applied:

(46) $$U_{i}\leftarrow U_{i}-W_{\mathrm{tot}}.$$

For each $U_{i}$, the interval $\left[W_{j},W_{j+1}\right]$ satisfying $W_{j}\leq U_{i}<W_{j+1}$ is located, and $t_{i}$ is recovered by linear interpolation:

(47) $$t_{i}=t_{j}+\frac{U_{i}-W_{j}}{W_{j+1}-W_{j}}\left(t_{j+1}-t_{j}\right).$$

The recovered parameters are sorted in ascending order, and the corresponding sampling points are then evaluated as

(48) $$P_{i}=r\left(t_{i}\right),\;i=1,2,\ldots ,N,$$

and connected in this parameter order to form the final sampling polygon.

**Figure 4:**
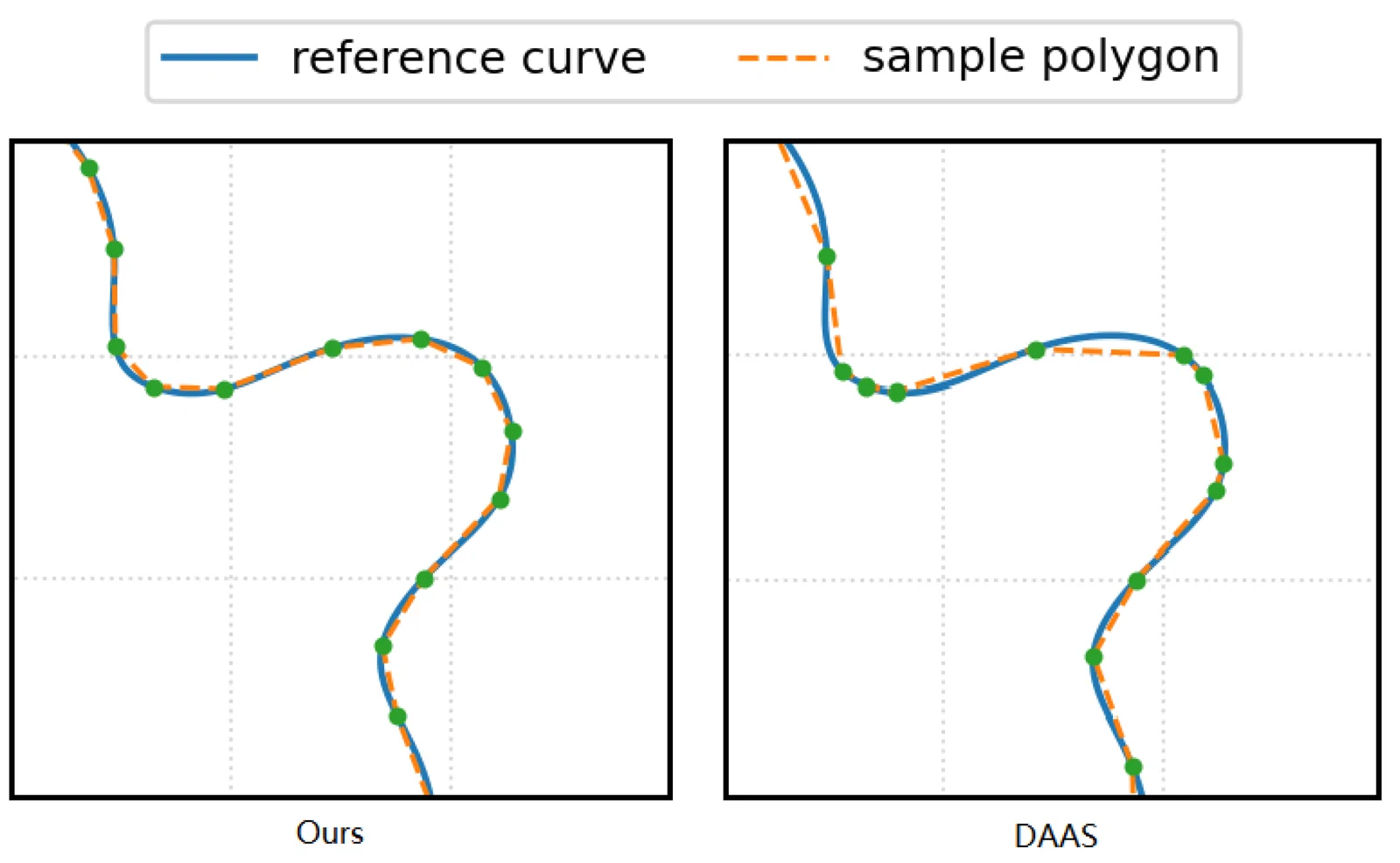
Local comparison between the proposed method and DAAS. Green dots indicate sampling points.

Accordingly, the method allocates more sampling points to regions with larger values of ${\left(\kappa \left(t\right)+\epsilon \right)}^{1/3}\left|r^{\prime }\left(t\right)\right|$ in the parameter domain, providing the regularized numerical realization of the theoretical cube-root-curvature density in the arc-length domain (Figure 4).

For reproducibility, Algorithm 1 summarizes the discrete realization used in all main experiments.
**Algorithm 1** Discrete realization of the area-driven sampling algorithm**Require:** Periodic fitted contour r(t), budget *N*, starting parameter $t_{0}$, numerical floor    $\epsilon =10^{-6}$ **Ensure:** Ordered samples ${\left\{P_{i}\right\}}_{i=1}^{N}$
 1:Set M←100N and construct nodes ${\left\{t_{j}\right\}}_{j=0}^{M}$ on [0,T] 2:Evaluate $r^{\prime }\left(t_{j}\right)$, $r^{\prime \prime }\left(t_{j}\right)$, $\kappa (t_{j})$, and $|r^{\prime }\left(t_{j}\right)|$ 3:Compute $w_{j}={\left(\kappa \left(t_{j}\right)+\epsilon \right)}^{1/3}\left|r^{\prime }\left(t_{j}\right)\right|$ 4:Compute $W_{j}$ by the trapezoidal recurrence in (43) 5:Set $W_{\mathrm{tot}}\leftarrow W_{M}$, $\Delta W\leftarrow W_{\mathrm{tot}}/N$, and obtain $W(t_{0})$ by interpolation 6:**for** i=1,2,…,N **do** 7:     $U_{i}\leftarrow W\left(t_{0}\right)+\left(i-1\right)\Delta W$ 8:     **if** $U_{i}\geq W_{\mathrm{tot}}$ **then** 9:        $U_{i}\leftarrow U_{i}-W_{\mathrm{tot}}$10:    **end if**11:    Locate *j* such that $W_{j}\leq U_{i}<W_{j+1}$ and recover $t_{i}$ by linear interpolation12:**end for**13:Sort ${\left\{t_{i}\right\}}_{i=1}^{N}$ in ascending parameter order and evaluate $P_{i}=r\left(t_{i}\right)$14:**return** ${\left\{P_{i}\right\}}_{i=1}^{N}$


## 4. Experimental Setup

### 4.1. Datasets and Reference Construction

**Random Closed Curves.** Random closed curves comprises 100 closed curves. For each random seed, 30 angular locations are placed on a unit circle, and the radial coordinate is perturbed using seven Fourier harmonics. The sine and cosine coefficients are independently drawn from a zero-mean Gaussian distribution with standard deviation 0.1, and the radial coordinate is lower-bounded by 0.3 to avoid degenerate shapes. Because the nominal radius is fixed at 1, all curves share a common geometric scale. The ordered points are fitted by a chord-length-parameterized periodic cubic spline without an additional smoothing parameter. A dense discretization of 10,000 spline points defines the reference contour, and its enclosed area is computed by the shoelace formula. The resulting benchmark provides smooth closed contours with varying local curvature and concavity for controlled geometric evaluation.

**Droplet Dataset.** The droplet dataset is an in-house, non-public dataset comprising 100 droplet images acquired in the OLED inkjet-printing droplet-observation setup at 2048×2048 pixels, with an imaging precision of 0.8 µm/pixel under high-intensity point-source illumination. Before contour extraction, the images undergo motion compensation and illumination equalization, followed by binary segmentation. The detailed implementation of these upstream procedures is proprietary and protected by patents. A dense ordered boundary is then extracted from each binary image and supplied to the contour-sampling stage. Subsequent contour fitting and reference-area construction follow the same procedure as for random closed curves, with the densely sampled fitted contour used to obtain $A_{\mathrm{ref}}$. Independent physical-area measurements are not available for this dataset; $A_{\mathrm{ref}}$ therefore provides the common reference for evaluating sampling-induced area error on the droplet dataset. Because the real acquisition dataset is internal and non-public, random closed curves provide a reproducible benchmark alongside the real-data evaluation.

### 4.2. Benchmarking Protocol

For every contour in random closed curves and the droplet dataset and every sampling budget N∈{20,40,60,80,100}, all methods receive the same fitted curve and return exactly *N* distinct samples in contour order. Connecting adjacent samples forms $P_{N}$, whose area is computed by the same shoelace implementation for all methods.

### 4.3. Compared Methods

Some compared methods were not originally formulated to return a prescribed number of sampling points. Table 1 therefore reports the fixed-*N* realization used for every method together with the numerical settings and stopping or final point-number adjustment required to obtain the common sampling budget *N*. For each baseline, the parameter values reported or recommended in the original publication are retained whenever they are directly applicable. When the fixed-*N* realization introduces additional numerical settings or requires adaptation of the original stopping rule, these settings are tuned under the fixed-*N* protocol to obtain stable performance while preserving the original optimization criterion. No parameter is selected using the reference area of the evaluated contour.

### 4.4. Evaluation Metrics and Statistical Analysis

#### 4.4.1. Area Estimation Metrics

The area estimation metrics include absolute area error, relative area error, mean absolute error (MAE), root mean square error (RMSE), and the coefficient of determination ($R^{2}$). These metrics evaluate the accuracy of the polygonal area obtained from the sampled contour relative to the reference area, which is the primary evaluation objective of this study.

For a fixed sampling budget, let *n* denote the number of evaluated contours. For contour *j*, the polygonal area $A_{N}^{\left(j\right)}$ is computed from $P_{N}$ using the shoelace formula, and the corresponding reference area is $A_{\mathrm{ref}}^{\left(j\right)}$. Here, $A_{\mathrm{ref}}^{\left(j\right)}$ is the dense numerical approximation of the fitted-contour area *A* in (4). The absolute and relative area errors are defined as

(49) $$E_{\mathrm{abs}}^{\left(j\right)}=\left|A_{N}^{\left(j\right)}-A_{\mathrm{ref}}^{\left(j\right)}\right|,\;E_{\mathrm{rel}}^{\left(j\right)}=100\;\frac{\left|A_{N}^{\left(j\right)}-A_{\mathrm{ref}}^{\left(j\right)}\right|}{\left|A_{\mathrm{ref}}^{\left(j\right)}\right|}.$$

For consistency with the notation used in several experimental figures, $A_{\mathrm{err}}$ denotes the same absolute area error as $E_{\mathrm{abs}}$ in (49).

The corresponding MAE and RMSE over the evaluated contours are

(50) $$\mathrm{MAE}=\frac{1}{n}\sum_{j=1}^{n}E_{\mathrm{abs}}^{\left(j\right)},\;\mathrm{RMSE}=\sqrt{\frac{1}{n}\sum_{j=1}^{n}{\left(A_{N}^{\left(j\right)}-A_{\mathrm{ref}}^{\left(j\right)}\right)}^{2}}.$$

The coefficient of determination is

(51) $$R^{2}=1-\frac{\sum_{j=1}^{n}{\left(A_{N}^{\left(j\right)}-A_{\mathrm{ref}}^{\left(j\right)}\right)}^{2}}{\sum_{j=1}^{n}{\left(A_{\mathrm{ref}}^{\left(j\right)}-{\bar{A}}_{\mathrm{ref}}\right)}^{2}},$$

where ${\bar{A}}_{\mathrm{ref}}$ is the mean reference area over the same set of contours. Absolute and relative area errors characterize the error of individual contours, while MAE, RMSE, and $R^{2}$ summarize area estimation performance over the evaluated contour set.

#### 4.4.2. Geometric Fidelity Metrics

The geometric fidelity metrics include Hausdorff distance, normalized mean contour distance (NMCD), maximum normalized contour deviation (NMCD_max_), and contour-fidelity RMS. These metrics quantify the geometric deviation between the sampling polygon and the fitted reference contour.

For one contour, let $r(s_{q})$, q=1,…,10,000 denote the dense reference points used to construct $A_{\mathrm{ref}}$. The shortest distance from each reference point to the boundary of $P_{N}$ is defined as

(52) $$d_{q}=\min_{y\in \partial P_{N}}{∥r(s_{q})-y∥}_{2}.$$

Let *L* denote the perimeter of the dense reference contour, corresponding to the total arc length in (3). NMCD, NMCD_max_, and contour-fidelity RMS are then computed as

(53) $$\mathrm{NMCD}=\frac{1}{10,000L}\sum_{q=1}^{10,000}d_{q},\;{\mathrm{NMCD}}_{\max}=\frac{\max_{q}d_{q}}{L},$$

and

(54) $$\mathrm{Fidelity}\;\mathrm{RMS}=\frac{1}{L}\sqrt{\frac{1}{10,000}\sum_{q=1}^{10,000}d_{q}^{2}}.$$

NMCD measures the average contour deviation, NMCD_max_ characterizes the largest one-sided contour deviation, and contour-fidelity RMS places greater weight on relatively large local deviations.

For the Hausdorff distance, the boundary of $P_{N}$ is densely resampled at points $y_{\ell }$. The quantity reported in the tables is the symmetric Hausdorff distance normalized by the same reference-contour perimeter:

(55) $$\mathrm{Hausdorff}=\frac{1}{L}\max\;\left\{\max_{q}\min_{\ell }{∥r\left(s_{q}\right)-y_{\ell }∥}_{2},\;\max_{\ell }\min_{q}{∥y_{\ell }-r\left(s_{q}\right)∥}_{2}\right\}.$$

The Hausdorff distance measures the largest bidirectional discrepancy between the reference contour and the sampling polygon. These measures follow the standard use of contour-deviation measures in polygonal-approximation evaluation [41].

#### 4.4.3. Statistical Analysis

Statistical analysis is performed on the per-contour area errors defined in Equation (49). For $E_{\mathrm{abs}}^{\left(j\right)}$ and $E_{\mathrm{rel}}^{\left(j\right)}$, the sample mean and standard deviation are reported to characterize the central tendency and variability across contours, and the corresponding 95% Student-*t* confidence intervals are additionally computed [42]. Because all methods are evaluated on the same contours, statistical comparisons against OURS are performed using paired values of $E_{\mathrm{abs}}^{\left(j\right)}$. Specifically, for each contour *j*, the difference between the $E_{\mathrm{abs}}^{\left(j\right)}$ of a compared method and that of OURS is evaluated using both the paired *t*-test and the two-sided Wilcoxon signed-rank test [42,43]. The Wilcoxon *p*-values obtained from the method-wise comparisons at each fixed sampling budget *N* are further adjusted using the Holm procedure [44], and $p_{\mathrm{Holm}}<0.05$ is regarded as statistically significant. For the overall tables, $E_{\mathrm{abs}}^{\left(j\right)}$ is first averaged over the tested sampling budgets for each contour, and the same paired tests are then applied to these per-contour mean errors; the *p*-values obtained separately at different *N* are not averaged.

## 5. Results

### 5.1. Sampling Visualization

The sampling visualization presents results on both simulated random closed curves and real droplet contours, comparing the point distributions produced by different sampling strategies under identical sampling budgets.

**Figure 5:**
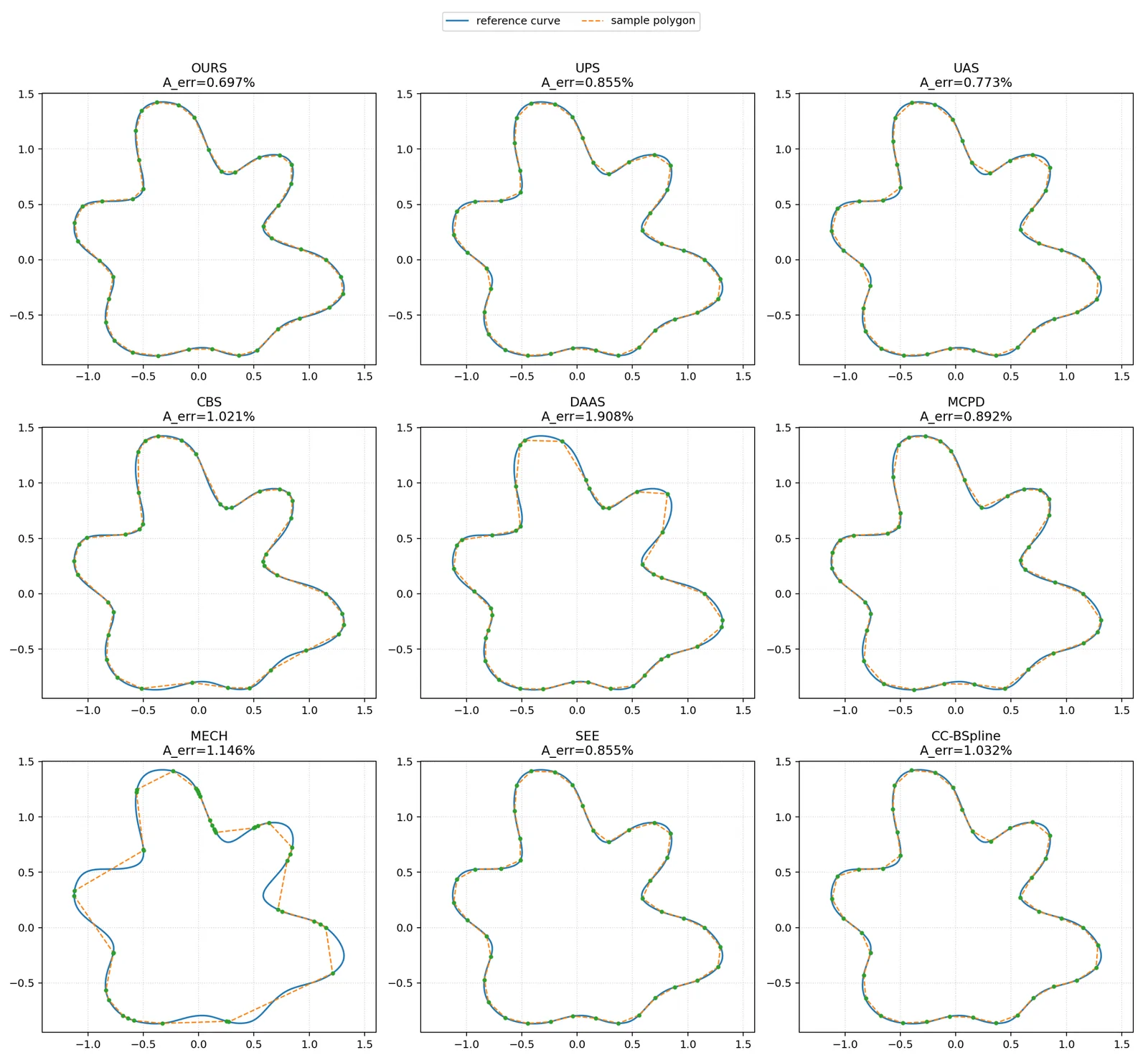
Example sampling results on a random closed curve. Green dots indicate sampling points.

**Figure 6:**
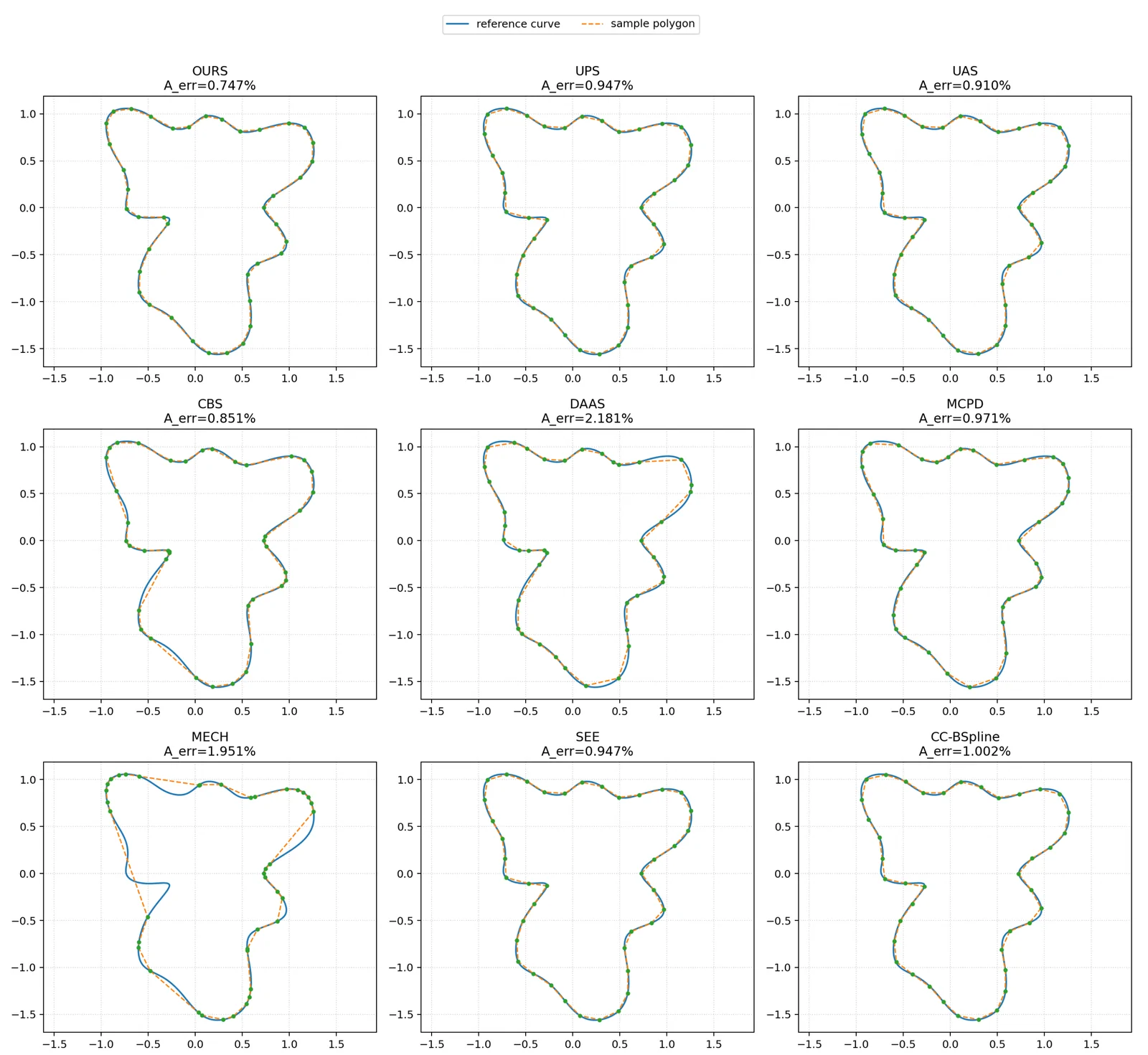
Example sampling results on another random closed curve. Green dots indicate sampling points.

**Figure 7:**
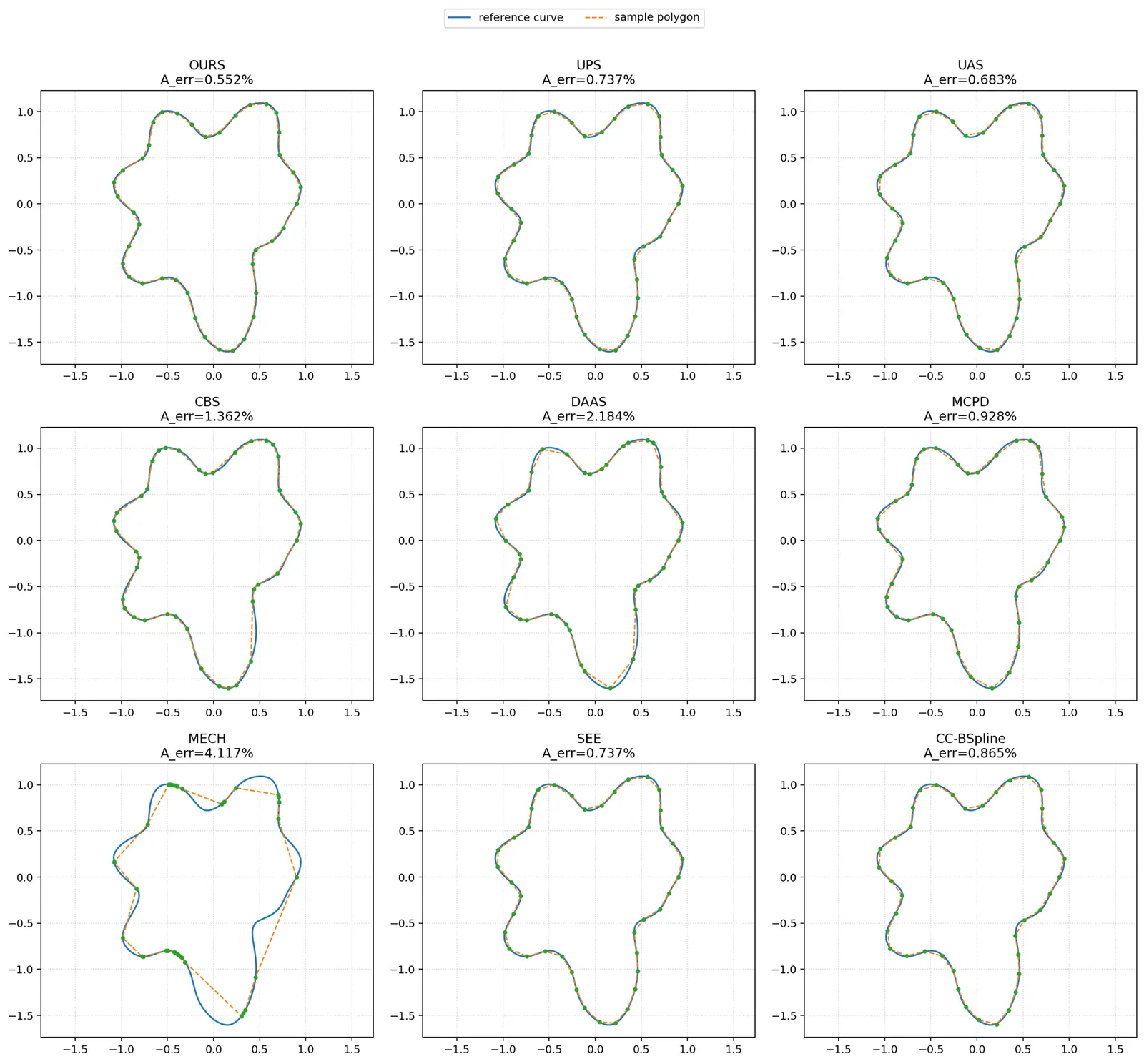
Example sampling results on yet another random closed curve. Green dots indicate sampling points.

**Random closed curves.** Figure 5, Figure 6 and Figure 7 present representative examples from the random closed-curve benchmark. The methods produce visibly different sampling-point distributions: UPS and UAS distribute points more uniformly along the contour, CBS and MECH allocate more points near high-curvature locations, and DAAS and MCPD respond more strongly to local geometric discrepancy. These allocation patterns are reflected in the resulting area estimation errors. The proposed area-driven sampling method distributes the sampling points according to the derived area-driven density. The visualization illustrates the resulting differences in sampling-point distribution, while geometric fidelity is assessed quantitatively in the Statistical Results subsection.

**Figure 8:**
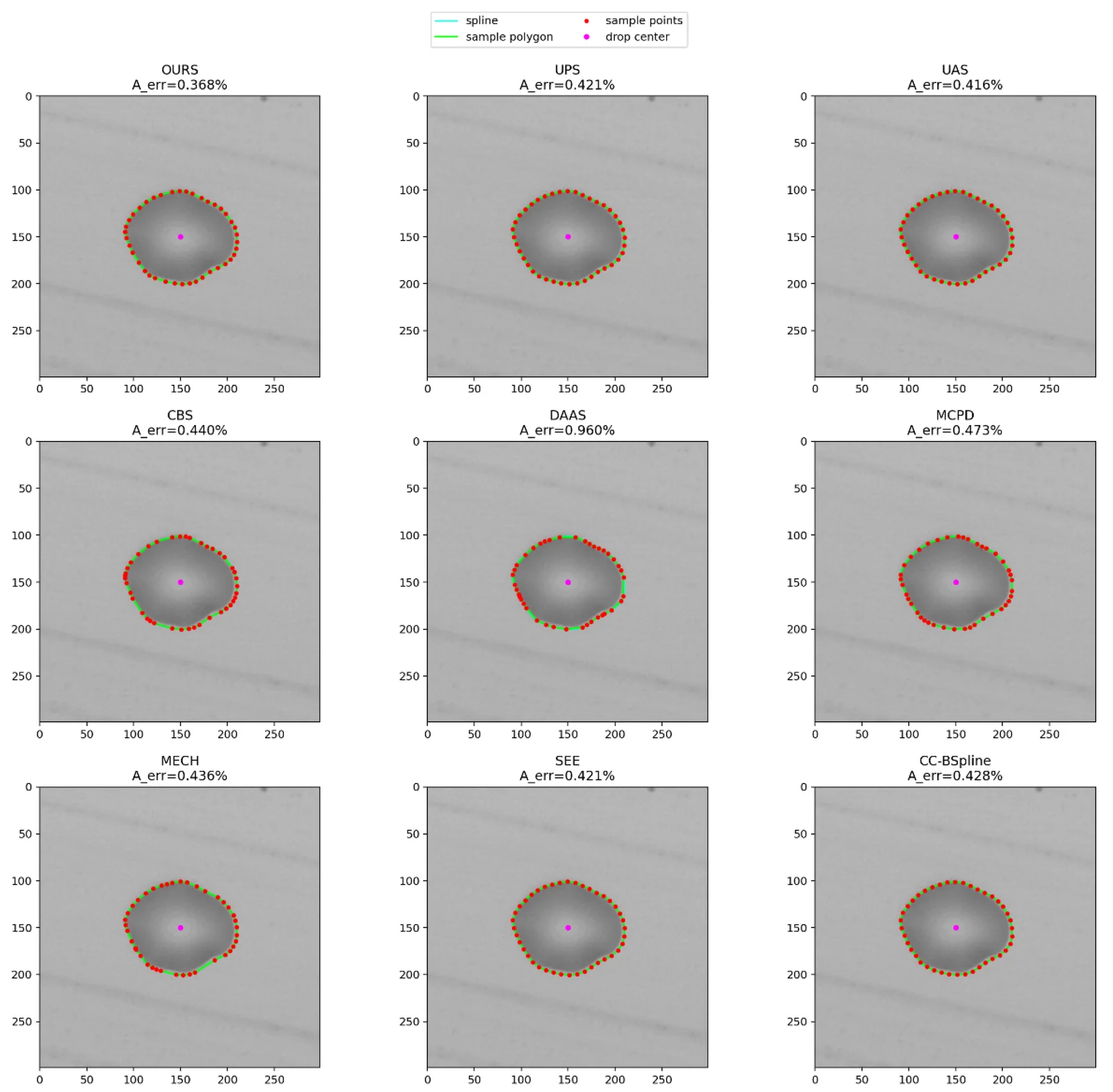
Example sampling results on a real droplet contour.

**Figure 9:**
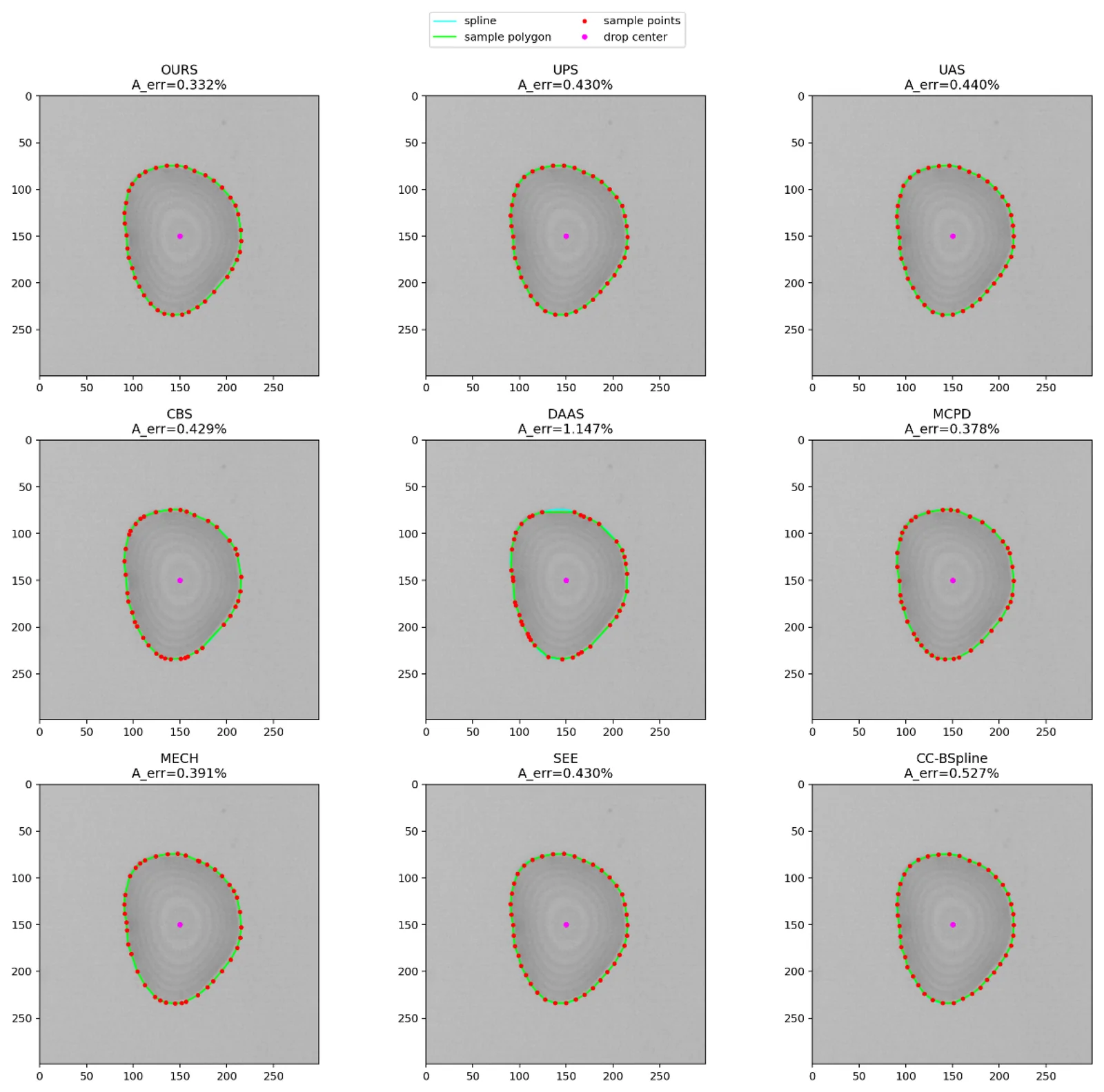
Example sampling results on a real droplet contour.

**Figure 10:**
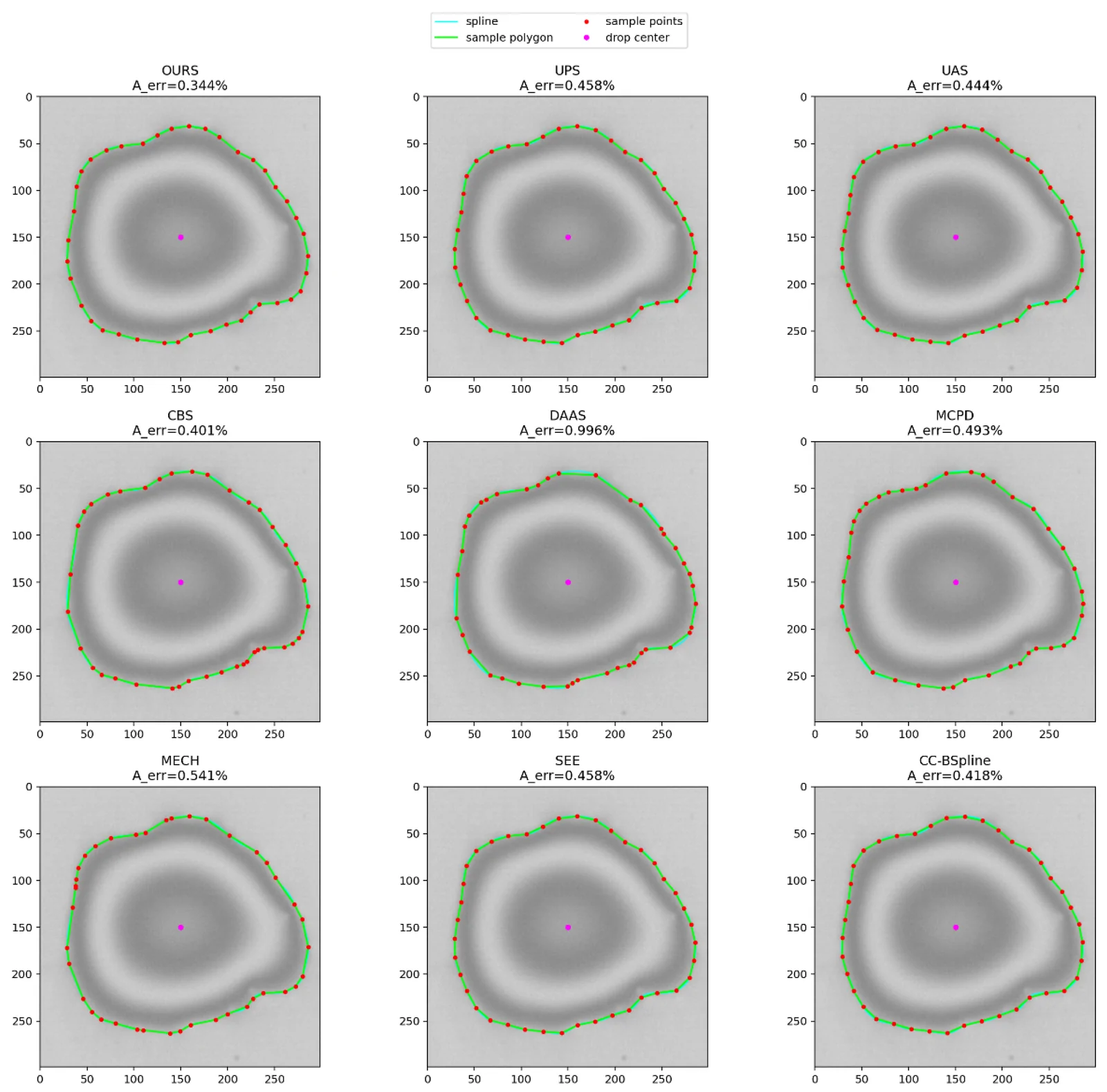
Example sampling results on a real droplet contour.

**Droplet Dataset.** Figure 8, Figure 9 and Figure 10 present representative examples from the real droplet dataset under a fixed sampling budget. In each panel, the cyan curve denotes the fitted contour, the green curve denotes the sampling polygon, the red markers indicate the sampled points, and the magenta marker indicates the droplet center. Similar differences in sampling-point allocation are observed: UPS and UAS remain comparatively uniform, CBS and MECH concentrate more samples near high-curvature locations, and DAAS and MCPD respond to local geometric discrepancy. These allocation patterns are reflected in the corresponding area estimation errors. The proposed area-driven sampling method allocates samples according to the derived area-driven density. Geometric fidelity is assessed quantitatively by Hausdorff distance, NMCD, NMCD_max_, and contour-fidelity RMS in the Statistical Results subsection.

### 5.2. Statistical Results

Table 2 and Table 3 report the results averaged over all tested sampling budgets, while Table 4 and Table 5 report the complete results for each *N*. The tables include area estimation accuracy, geometric-fidelity metrics, and paired statistical tests. For random closed curves, OURS gives the lowest overall MAE (0.0234), RMSE (0.0244), and mean relative error (0.7130%). For the droplet dataset, OURS gives the lowest overall MAE (141.5260), RMSE (161.9166), and mean relative error (0.4410%). The per-budget results show that the absolute difference in area estimation error is largest at lower sampling budgets and decreases as *N* increases.

The paired *t*-test, Wilcoxon signed-rank test, and Holm-corrected *p*-values are reported directly in the detailed tables for each sampling budget. The geometric-fidelity metrics provide a complementary assessment of contour degradation. Across the reported results, the reduction in area estimation error is not accompanied by a systematic or disproportionate loss of contour fidelity.

### 5.3. Accumulated Local Area Error

The accumulated-local-area-error analysis connects the local arc–chord area terms in the leading-order analysis with their empirical distribution along the contour. The cumulative absolute local error serves as a diagnostic of local error accumulation, complementary to the global polygonal area error $E_{N}$ in (8).

**Figure 11:**
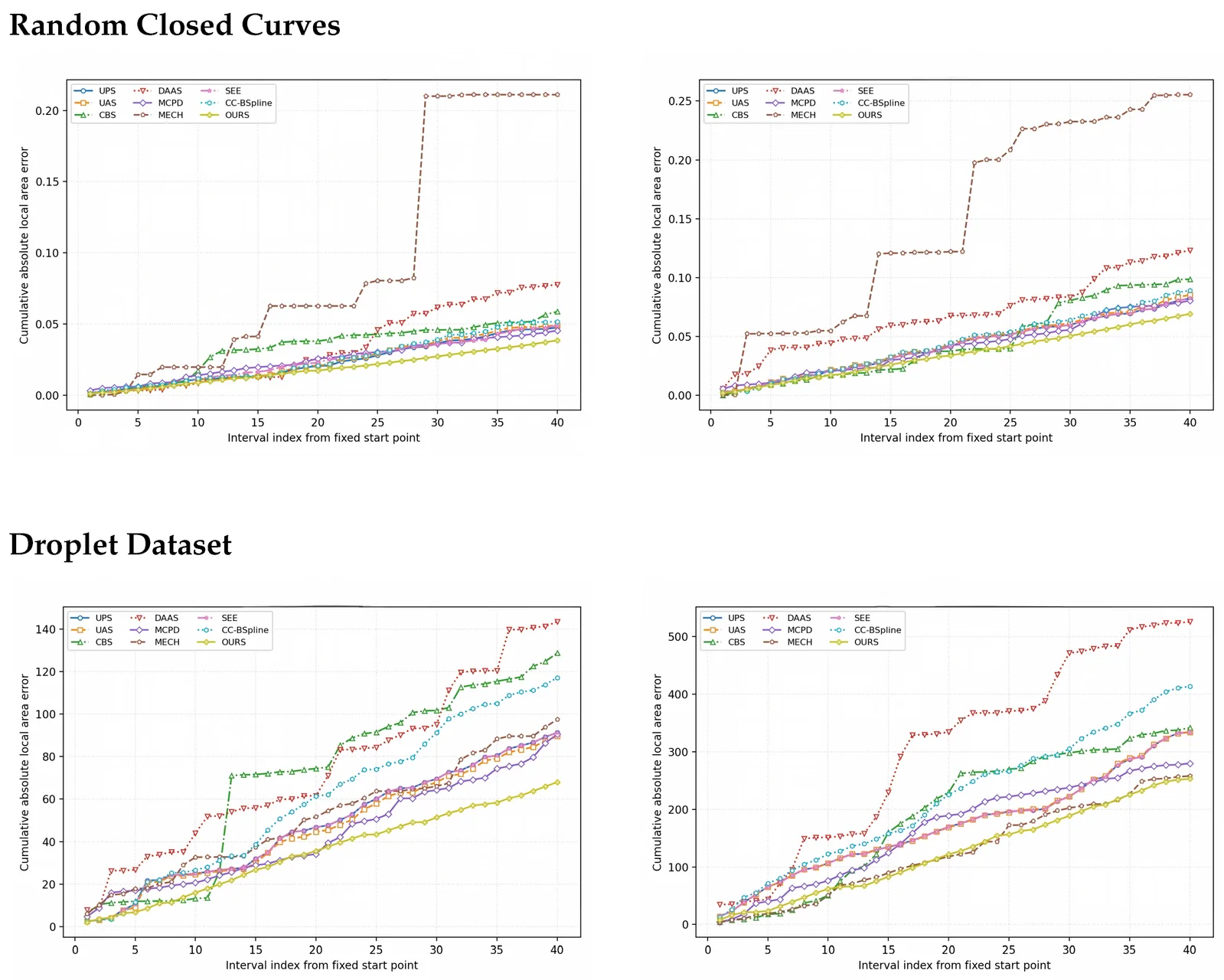
Accumulated local arc–chord area error under a fixed sampling budget. The top row shows two random closed curves, and the bottom row shows two real droplet contours. The proposed area-driven sampling method reduces local error accumulation more effectively.

For each sampling interval, the absolute area discrepancy enclosed by the contour arc and its corresponding chord is computed and accumulated along the contour traversal. Figure 11 presents representative results for random closed curves and the droplet dataset. In both cases, the proposed area-driven sampling method produces a lower cumulative local error than several compared methods. Its cumulative-error curve also exhibits a more gradual growth over the contour, which is consistent with a more even distribution of the local arc–chord area contributions among the sampling intervals. These observations complement the global area-error results by showing how the local errors underlying the leading-order analysis are distributed along the contour.

### 5.4. Sampling-Budget Experiment Results

The sampling-budget experiment evaluates whether Corollary 1 can provide a practically useful sampling-budget estimate under a target area-error tolerance. Unlike the simulated benchmark, the droplet dataset contains contours with varying sizes and shapes. For this reason, the estimation is performed under a *relative* accuracy requirement, allowing the target tolerance to adapt to the geometric scale of each droplet.

For each droplet contour, a high-density discretization was first used to obtain a reference area $A_{\mathrm{ref}}$ and the integral curvature required by (37). Given a target relative area-error tolerance τ∈{0.1%,0.2%,…,1.0%}, we converted it into the corresponding absolute tolerance using $\varepsilon_{\mathrm{abs}}=\frac{\tau A_{\mathrm{ref}}}{100\%}$. Then, we estimated the required number of sampling points via (37), and measured the actual relative area error after discretization with the estimated budget. Table 6 reports the mean and standard deviation of the estimated sample size, the mean and standard deviation of the achieved relative area error, and the satisfaction rate, defined as the proportion of droplet contours for which the actual error does not exceed the prescribed target.

The estimated sampling budget increases as the target tolerance becomes stricter. The achieved relative area error remains close to the requested tolerance, and the satisfaction rate remains high across the tested range. This behavior is consistent with the leading-order sampling-budget estimation in (37).

## 6. Ablation Studies

### 6.1. AREA-GREEDY-LOCAL Baseline

AREA-GREEDY-LOCAL is included as an area-aware greedy reference under the same fitted-contour and fixed-budget constraints. This comparison evaluates whether the curvature-derived sampling law provides additional benefit beyond the use of an area-related sampling criterion itself. For a sampling budget *N*, an unselected candidate $P_{j}$, let $P_{i}$ and $P_{k}$ be its two currently selected neighbors along the closed contour. The local score is the absolute area of the triangle formed by these three points,

(56) $$\begin{array}{cc} G_{j} & =\frac{1}{2}\left|\det\;\left(P_{j}-P_{i},\;P_{k}-P_{i}\right)\right|\\ \; & =\frac{1}{2}\left|\left(x_{j}-x_{i}\right)\left(y_{k}-y_{i}\right)-\left(y_{j}-y_{i}\right)\left(x_{k}-x_{i}\right)\right|. \end{array}$$

This is the magnitude of the polygon-area increment produced when the current chord $P_{i}P_{k}$ is replaced by $P_{i}P_{j}$ and $P_{j}P_{k}$. The next candidate is therefore selected by

(57) $$j^{★}=\underset{P_{j}\;\mathrm{unselected}}{\arg\max}\;G_{j}.$$

**Figure 12:**
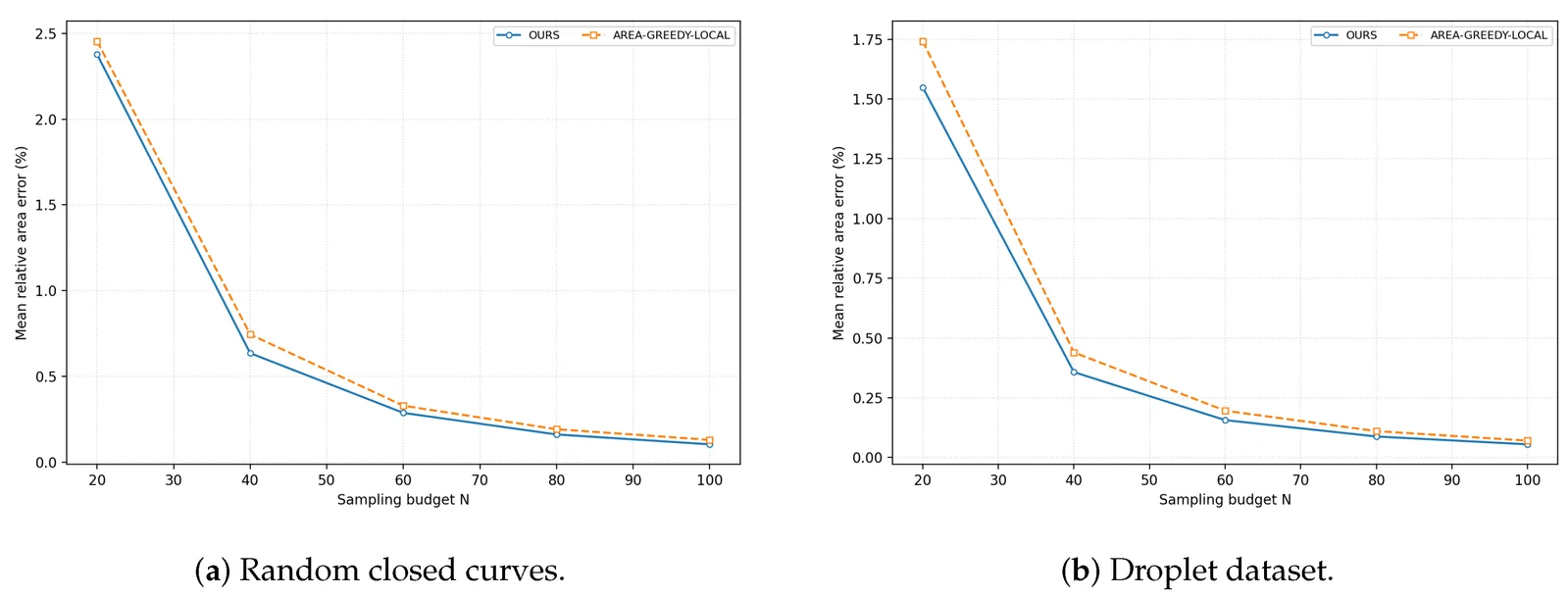
Comparison between OURS and AREA-GREEDY-LOCAL.

After insertion, the neighboring selected points and only the affected local scores are recomputed. The procedure continues until exactly *N* points are selected. AREA-GREEDY-LOCAL is thus a direct iterative local greedy strategy under the same fitted-contour and fixed-budget constraints. Figure 12 evaluates whether the analytically derived area-driven sampling density yields lower area estimation error than this local greedy strategy.

### 6.2. α Ablation

**Figure 13:**
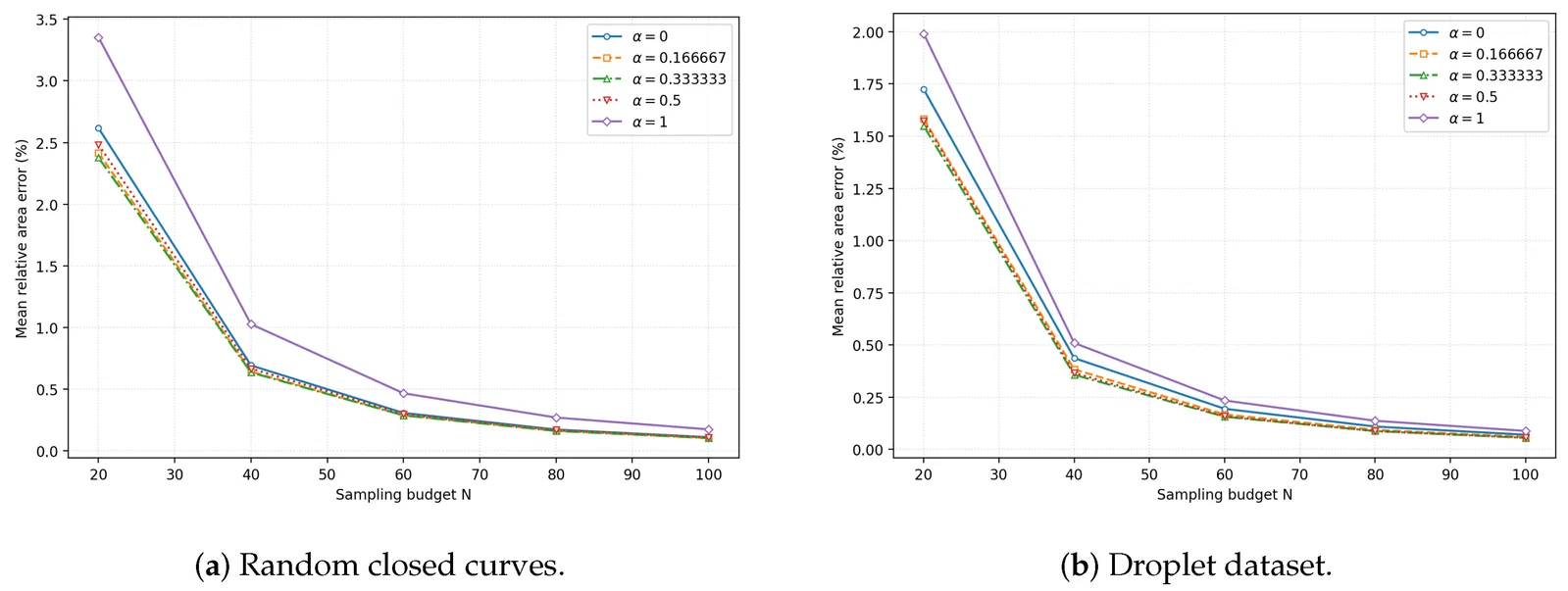
Ablation study for different α.

The exponent α=1/3 in (41) is further evaluated by replacing it with α∈{0,1/6,1/3,1/2,1}. Under this implementation, α=0 corresponds to arc-length weighting, whereas α=1/3 is the value derived from the leading-order area-error model. As shown in Figure 13, α=1/3 yields the lowest area estimation error among the tested exponents on both random closed curves and the droplet dataset. Deviating from 1/3 in either direction leads to higher error, providing empirical support for the curvature exponent derived from the leading-order analysis.

### 6.3. ϵ Sensitivity

**Figure 14:**
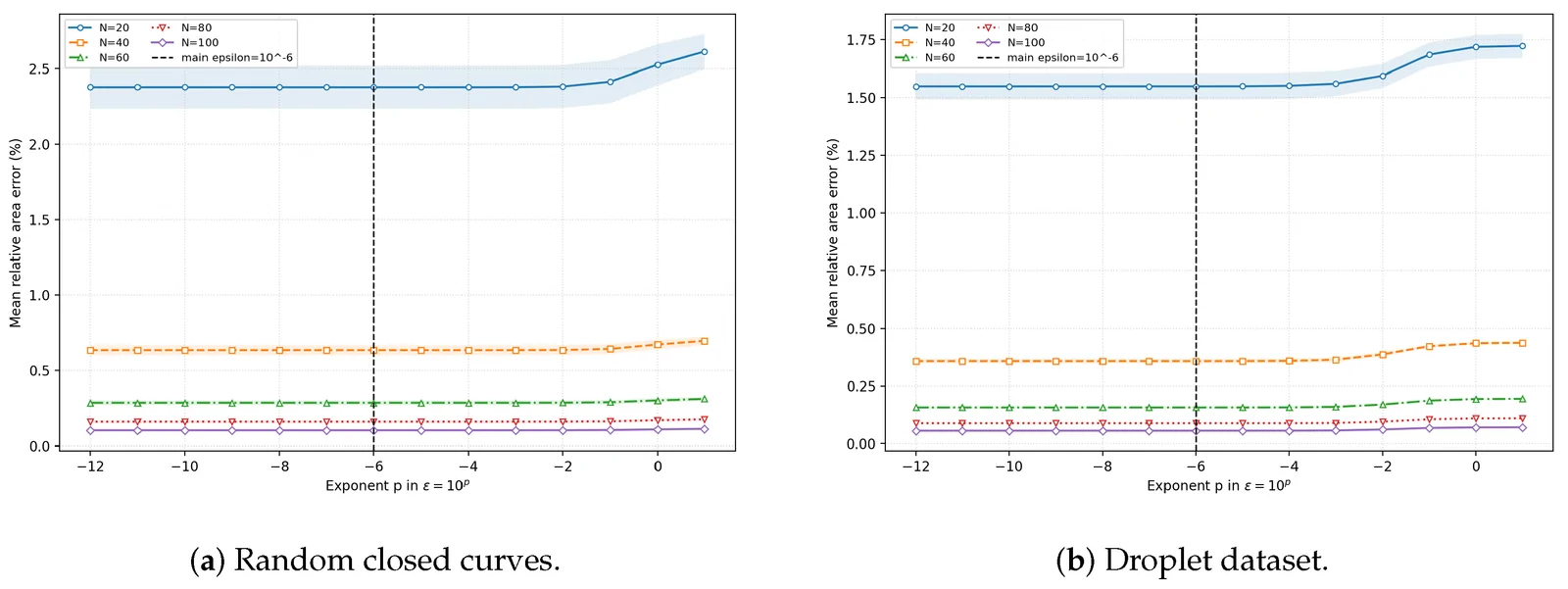
Sensitivity to ϵ over a logarithmic range.

The main experiments use $\epsilon =10^{-6}$ in (41). To characterize the influence of this numerical floor, ϵ is varied over integer powers of ten from $10^{-12}$ to $10^{1}$. As shown in Figure 14, the area estimation error remains essentially unchanged over the broad range from $10^{-12}$ to $10^{-2}$ on both random closed curves and the droplet dataset. When ϵ is increased beyond this range, the area estimation error gradually increases because the curvature-dependent weighting is increasingly weakened by the additive floor. These results indicate that sufficiently small values of ϵ have negligible influence on the sampling distribution and that ϵ primarily serves as a numerical stabilization term for zero- or near-zero-curvature regions. The value $\epsilon =10^{-6}$ used in the main experiments lies well within this stable range.

### 6.4. Noise and Spline-Smoothing Sensitivity

**Figure 15:**
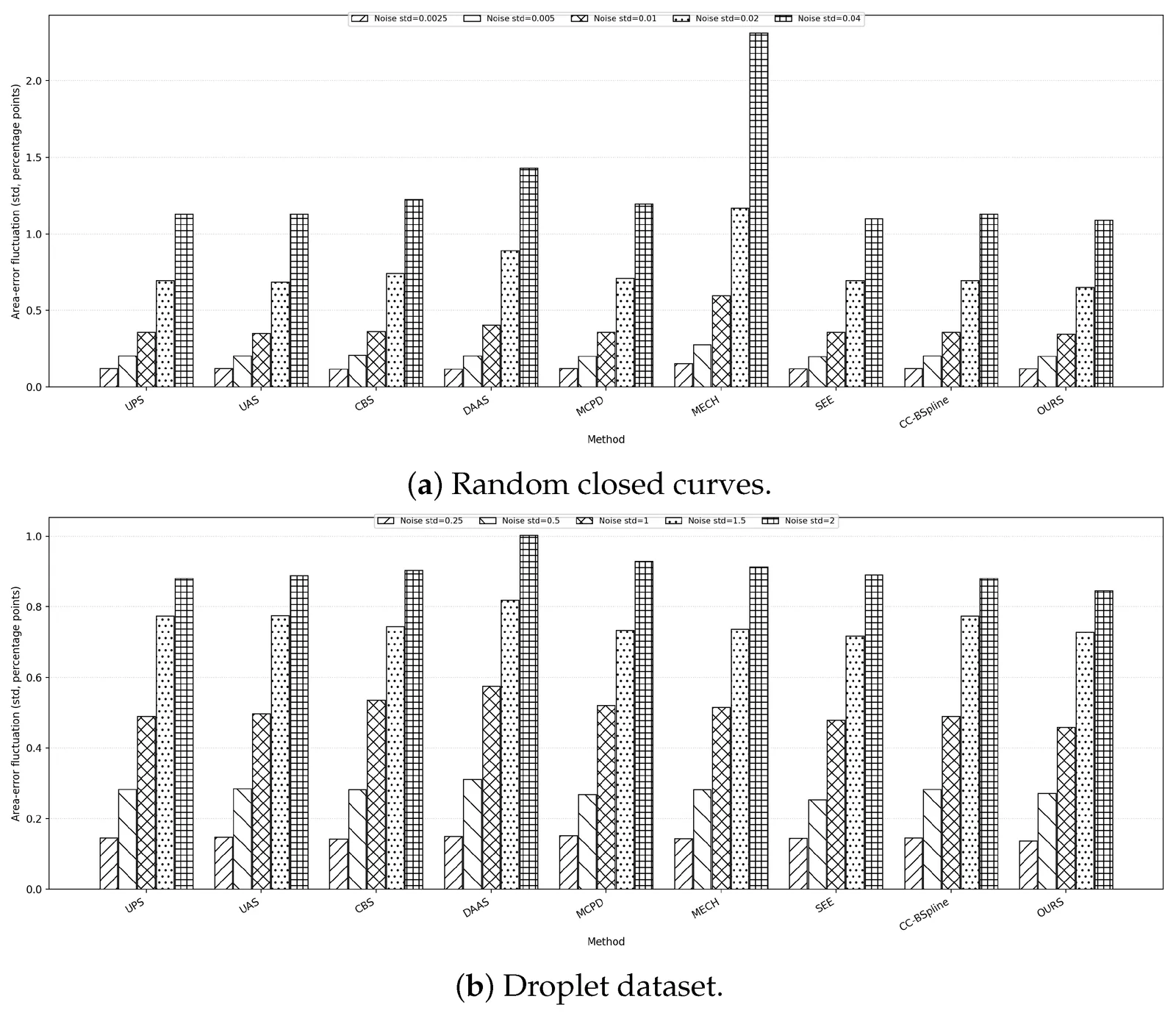
Noise-induced area-error fluctuation for multiple noise standard deviations and spline-smoothing ratios.

Noise and imaging uncertainties, including optical noise, motion blur, and segmentation error, are important practical issues in vision-based droplet observation. To emulate their downstream effects on the extracted contour, controlled contour perturbations and different spline-smoothing levels are introduced, and the resulting sensitivity of the sampling methods is evaluated. This approximation is motivated by the fact that these imaging and segmentation uncertainties ultimately manifest as spatial deviations of the extracted boundary, while spline smoothing controls how such deviations are propagated into the fitted contour. Specifically, the reference contour is perturbed using multiple noise standard deviations and then refitted with several spline-smoothing ratios. Results are averaged over the tested sampling budgets, and Figure 15 reports the resulting area-error fluctuations.

As the simulated degradation becomes stronger, the area estimation error generally increases for all sampling methods, including OURS, with broadly similar variation trends across the compared strategies. Changes in the spline-smoothing ratio affect the magnitude of the error fluctuation, but no method exhibits a distinctly different response to the simulated perturbations. These results show that OURS follows a disturbance-response pattern comparable to those of the other sampling methods under the contour deviations used to approximate the downstream effects of practical imaging and segmentation uncertainties.

## 7. Runtime Analysis

**Figure 16:**
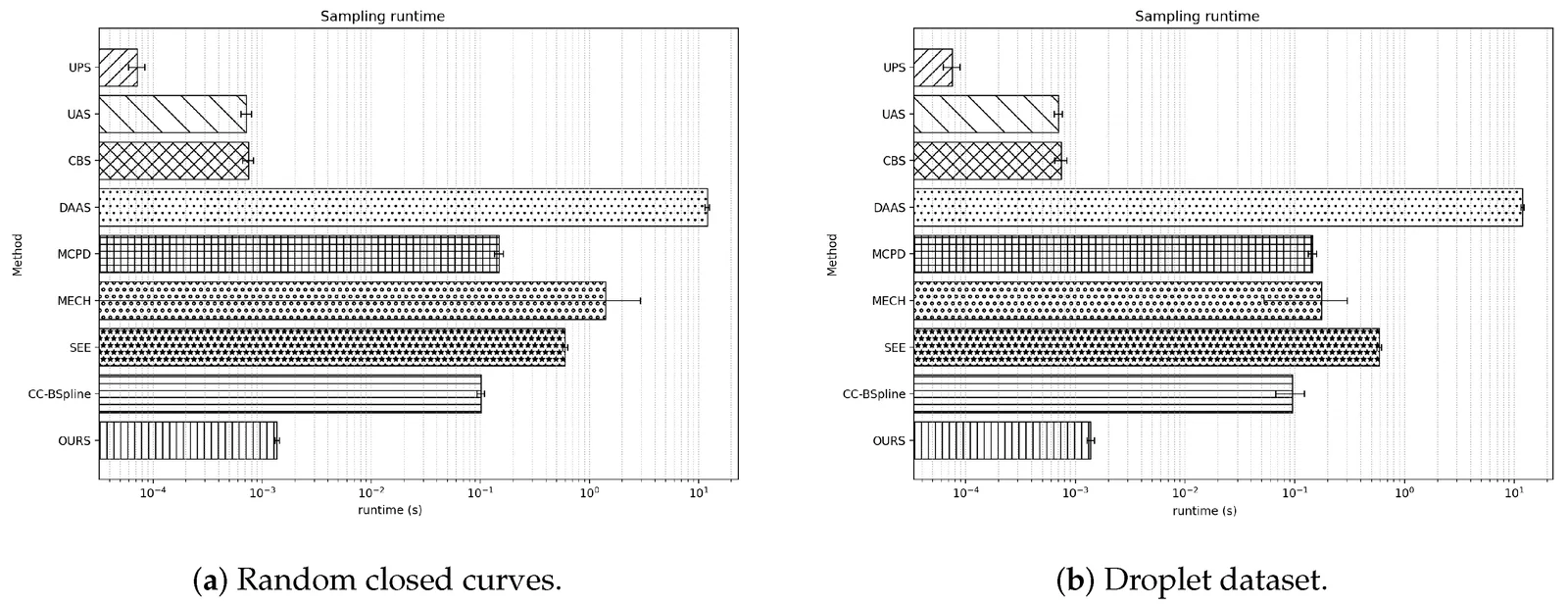
Runtime comparison of the sampling methods. Methods are shown on the vertical axis; the central marker and whiskers summarize the recorded runtime distribution across contours.

Runtime is evaluated to quantify the computational cost of each sampling strategy under the same experimental conditions. For every contour and sampling budget, only the execution time of the sampling procedure is recorded, while data loading, spline fitting, metric computation, plotting, and file I/O are excluded. Figure 16 summarizes the runtime distributions on random closed curves and the droplet dataset.

The proposed method introduces additional computation for curvature evaluation and cumulative-weight inversion. Its computational cost is assessed jointly with the corresponding improvement in area-estimation accuracy. Considering the reduction in area estimation error achieved under the same sampling budget, the additional runtime is relatively limited and represents a practical accuracy–computation trade-off for applications in which area-estimation accuracy is the primary requirement.

## 8. Conclusions

This work presents an area-driven sampling method for closed droplet contours in vision-based droplet observation. Starting from the leading-order local arc–chord area error, the derived global error model leads to a sampling density proportional to the cube root of curvature. The resulting optimality is interpreted asymptotically with respect to the leading-order error functional, while the expression under a prescribed area-error tolerance provides a leading-order estimate of the required sampling budget.

Experiments on random closed curves and the droplet dataset demonstrate that the proposed method consistently reduces polygonal area estimation error under the tested sampling budgets, without a disproportionate degradation of contour fidelity. The improvement is particularly evident at lower sampling budgets, indicating that the proposed sampling strategy is most useful when only a limited number of contour points can be retained. This property is relevant to high-frame-rate droplet observation and other applications in which contour representation is constrained by storage, transmission, or computational resources.

The proposed method is centered on area-driven sampling of a fitted, single closed droplet contour. A natural next step is to integrate the sampling strategy into a complete vision-based measurement pipeline so that the effects of image acquisition, segmentation, calibration, and physical-reference uncertainty can be evaluated jointly. Extending the method to more complex droplet configurations, such as multiple, interacting, merging, or breaking droplets, is another important direction and will require coupling the sampling strategy with contour detection and tracking. These extensions would further broaden the applicability of area-driven contour sampling in practical droplet-observation systems.

## Figures and Tables

**Table 1:** Implementation of the compared methods under the common fixed-budget protocol.

Method	Original Criterion	Fixed-*N* Realization	Main Numerical Settings
UPS	Uniform parameter spacing	Directly evaluate *N* equally spaced parameters on the periodic spline.	$t_{0}=0$.
UAS	Uniform arc-length spacing	Numerically invert cumulative arc length at *N* equal arc-length quantiles.	Arc-length grid: 100N.
CBS	Curvature allocation	Build cumulative curvature weight and invert it at *N* equal weight quantiles.	α=1, $\epsilon =10^{-6}$.
DAAS	Reconstruction deviation	Initialize an oversampled polygon and greedily remove the point whose deletion produces the smallest reconstruction-deviation penalty until *N* remain.	Initial count max(3N,N+16).
MCPD	Maximum corresponding deviation	Initialize with ⌈3N/4⌉ points and repeatedly insert the location of maximum interval deviation until *N* are obtained.	-.
MECH	Equal chord-height threshold	Search the threshold by binary search to approach the requested cardinality; if necessary, apply the smallest insertion/removal adjustment to return exactly *N* points.	ω=1.5, p=0.6, q=1.5, r=0.5, control factor m=5.
SEE	Sectional-error equivalence	Maintain N+1 open-interval parameters, iteratively equalize sectional errors, and remove the duplicated periodic endpoint to return *N* distinct samples.	$\epsilon_{\mathrm{var}}=10^{-10}$; max. 120 iterations.
CC-BSpline	Concavity/convexity reconstruction	Extract concavity/convexity control points, reconstruct a periodic B-spline, and evaluate exactly *N* parameters on the reconstructed spline.	Raster size 256×256; $L_{1}=L_{2}=2$; smoothing 0.
OURS	Area-driven density	Direct fixed-budget cumulative-weight inversion of the proposed density.	$\epsilon =10^{-6}$, $t_{0}=0$.

**Table 2:** Overall results of random closed curves, averaged over all tested sampling budgets.

Method	MAE	RMSE	$R^{2}$	Rel. Err. (%)	Abs. Err.	Hausdorff	NMCD	NMCD_max_	Fidelity RMS	$p_{t}$	$p_{W}$	$p_{\mathrm{Holm}}$	Sig.
UPS	0.0274	0.0282	0.2603	0.8377 ± 1.0339	0.0274 ± 0.0340	0.00399 ± 0.00440	0.00111 ± 0.00135	0.00398 ± 0.00441	0.00142 ± 0.00171	1.135 × $10^{-23}$	1.961 × $10^{-16}$	9.803 × $10^{-16}$	Yes
UAS	0.0255	0.0261	0.3493	0.7799 ± 0.9749	0.0255 ± 0.0321	0.00468 ± 0.00455	0.00115 ± 0.00139	0.00468 ± 0.00456	0.00151 ± 0.00177	2.945 × $10^{-9}$	8.452 × $10^{-9}$	8.452 × $10^{-9}$	Yes
CBS	0.0347	0.0388	−0.2976	1.0583 ± 1.4037	0.0347 ± 0.0463	0.00339 ± 0.00350	0.00101 ± 0.00111	0.00338 ± 0.00351	0.00127 ± 0.00139	2.298 × $10^{-16}$	2.292 × $10^{-14}$	9.168 × $10^{-14}$	Yes
DAAS	0.0555	0.0595	−1.1993	1.6928 ± 1.5460	0.0555 ± 0.0510	0.00660 ± 0.00519	0.00139 ± 0.00114	0.00660 ± 0.00519	0.00201 ± 0.00164	1.450 × $10^{-44}$	3.897 × $10^{-18}$	3.117 × $10^{-17}$	Yes
MCPD	0.0283	0.0306	0.1819	0.8641 ± 1.1006	0.0283 ± 0.0362	0.00353 ± 0.00431	0.00108 ± 0.00132	0.00352 ± 0.00431	0.00139 ± 0.00172	1.681 × $10^{-11}$	3.273 × $10^{-10}$	6.546 × $10^{-10}$	Yes
MECH	0.2995	0.4822	−94.7978	9.1577 ± 12.1527	0.2995 ± 0.3974	0.03407 ± 0.02762	0.00942 ± 0.01108	0.03407 ± 0.02762	0.01331 ± 0.01393	2.196 × $10^{-13}$	3.897 × $10^{-18}$	3.117 × $10^{-17}$	Yes
SEE	0.0336	0.0352	−0.3333	1.0258 ± 1.4596	0.0336 ± 0.0481	0.00404 ± 0.00458	0.00110 ± 0.00134	0.00403 ± 0.00458	0.00142 ± 0.00171	1.921 × $10^{-32}$	3.897 × $10^{-18}$	3.117 × $10^{-17}$	Yes
CC-BSpline	0.0273	0.0285	0.2980	0.8333 ± 0.9934	0.0273 ± 0.0327	0.00483 ± 0.00449	0.00128 ± 0.00134	0.00482 ± 0.00450	0.00164 ± 0.00171	3.634 × $10^{-12}$	1.753 × $10^{-10}$	5.260 × $10^{-10}$	Yes
OURS	0.0234	0.0244	0.4357	0.7130 ± 0.9159	0.0234 ± 0.0302	0.00273 ± 0.00348	0.00094 ± 0.00115	0.00271 ± 0.00349	0.00113 ± 0.00141	–	–	–	Ref.

Relative and absolute errors are reported as mean ± standard deviation. The paired *t*-test, Wilcoxon signed-rank test, and Holm-correctedWilcoxon *p*-value compare each method with OURS using absolute area error; “Ref.” denotes the reference method.

**Table 3:** Overall results of the droplet dataset, averaged over all tested sampling budgets.

Method	MAE	RMSE	$R^{2}$	Rel. Err. (%)	Abs. Err.	Hausdorff	NMCD	NMCD_max_	Fidelity RMS	$p_{t}$	$p_{W}$	$p_{\mathrm{Holm}}$	Sig.
UPS	162.4855	184.2260	0.9996	0.5098 ± 0.6360	162.4855 ± 242.8877	0.00228 ± 0.00245	0.00057 ± 0.00068	0.00227 ± 0.00245	0.00075 ± 0.00088	4.117 × $10^{-9}$	2.734 × $10^{-9}$	9.318 × $10^{-9}$	Yes
UAS	160.8101	181.6534	0.9997	0.5070 ± 0.6301	160.8101 ± 239.0312	0.00244 ± 0.00249	0.00058 ± 0.00068	0.00243 ± 0.00250	0.00077 ± 0.00089	2.634 × $10^{-8}$	3.011 × $10^{-8}$	6.023 × $10^{-8}$	Yes
CBS	196.4927	233.9673	0.9994	0.5919 ± 0.7551	196.4927 ± 310.3627	0.00208 ± 0.00224	0.00059 ± 0.00069	0.00206 ± 0.00225	0.00076 ± 0.00088	2.288 × $10^{-11}$	3.751 × $10^{-12}$	2.625 × $10^{-11}$	Yes
DAAS	374.9878	449.8409	0.9980	1.1594 ± 1.3836	374.9878 ± 587.8343	0.00523 ± 0.00509	0.00102 ± 0.00114	0.00522 ± 0.00509	0.00157 ± 0.00175	1.807 × $10^{-17}$	1.120 × $10^{-12}$	8.958 × $10^{-12}$	Yes
MCPD	154.7730	177.0015	0.9997	0.4936 ± 0.6220	154.7730 ± 225.8539	0.00182 ± 0.00208	0.00054 ± 0.00064	0.00179 ± 0.00210	0.00070 ± 0.00083	5.166 × $10^{-4}$	8.525 × $10^{-5}$	8.525 × $10^{-5}$	Yes
MECH	584.9636	1571.2311	0.9621	1.4962 ± 6.5407	584.9636 ± 2956.6665	0.00460 ± 0.01704	0.00143 ± 0.00674	0.00457 ± 0.01705	0.00189 ± 0.00866	0.00548	2.092 × $10^{-9}$	9.318 × $10^{-9}$	Yes
SEE	212.4792	248.1136	0.9992	0.6574 ± 0.9771	212.4792 ± 375.6272	0.00271 ± 0.00338	0.00067 ± 0.00090	0.00269 ± 0.00339	0.00090 ± 0.00121	3.375 × $10^{-13}$	4.677 × $10^{-12}$	2.806 × $10^{-11}$	Yes
CC-BSpline	176.4891	204.6381	0.9996	0.5563 ± 0.6434	176.4891 ± 248.4075	0.00280 ± 0.00232	0.00079 ± 0.00062	0.00279 ± 0.00233	0.00098 ± 0.00080	6.250 × $10^{-11}$	1.864 × $10^{-9}$	9.318 × $10^{-9}$	Yes
OURS	141.5260	161.9166	0.9997	0.4410 ± 0.5744	141.5260 ± 221.6533	0.00152 ± 0.00192	0.00050 ± 0.00062	0.00147 ± 0.00194	0.00060 ± 0.00077	–	–	–	Ref.

Relative and absolute errors are reported as mean ± standard deviation. The paired *t*-test, Wilcoxon signed-rank test, and Holm-correctedWilcoxon *p*-value compare each method with OURS using absolute area error; “Ref.” denotes the reference method.

**Table 4:** Per-budget results of random closed curves.

Method	*N*	MAE	RMSE	$R^{2}$	Rel. Err. (%)	Abs. Err.	Haus.	NMCD	NMCD_max_	Fid. RMS	$p_{t}$	$p_{W}$	$p_{\mathrm{Holm}}$	Sig.
UPS	20	0.0908	0.0933	−2.3707	2.7718 ± 0.6168	0.0908 ± 0.0214	0.01221 ± 0.00233	0.00366 ± 0.00065	0.01221 ± 0.00233	0.00467 ± 0.00078	5.734 × $10^{-16}$	4.682 × $10^{-13}$	2.341 × $10^{-12}$	Yes
UAS	20	0.0858	0.0878	−1.9865	2.6177 ± 0.5434	0.0858 ± 0.0190	0.01290 ± 0.00278	0.00377 ± 0.00072	0.01290 ± 0.00278	0.00484 ± 0.00089	6.766 × $10^{-7}$	8.345 × $10^{-7}$	1.669 × $10^{-6}$	Yes
CBS	20	0.1101	0.1218	−4.7505	3.3541 ± 1.5683	0.1101 ± 0.0525	0.00969 ± 0.00253	0.00307 ± 0.00059	0.00969 ± 0.00253	0.00385 ± 0.00079	1.724 × $10^{-11}$	2.398 × $10^{-10}$	9.593 × $10^{-10}$	Yes
DAAS	20	0.1378	0.1489	−7.5831	4.2032 ± 1.6949	0.1378 ± 0.0567	0.01536 ± 0.00557	0.00347 ± 0.00078	0.01536 ± 0.00557	0.00493 ± 0.00143	1.405 × $10^{-22}$	1.149 × $10^{-17}$	8.040 × $10^{-17}$	Yes
MCPD	20	0.0890	0.0971	−2.6535	2.7151 ± 1.1728	0.0890 ± 0.0391	0.01157 ± 0.00270	0.00356 ± 0.00082	0.01157 ± 0.00270	0.00459 ± 0.00108	5.323 × $10^{-4}$	5.423 × $10^{-4}$	5.423 × $10^{-4}$	Yes
MECH	20	0.4739	0.6214	−148.5822	14.4957 ± 12.3598	0.4739 ± 0.4040	0.04970 ± 0.02366	0.01458 ± 0.01068	0.04970 ± 0.02366	0.02014 ± 0.01303	2.895 × $10^{-16}$	2.964 × $10^{-17}$	1.779 × $10^{-16}$	Yes
SEE	20	0.1216	0.1279	−5.3350	3.7074 ± 1.1696	0.1216 ± 0.0399	0.01249 ± 0.00298	0.00363 ± 0.00066	0.01248 ± 0.00298	0.00466 ± 0.00086	7.213 × $10^{-28}$	6.125 × $10^{-18}$	4.900 × $10^{-17}$	Yes
CC-BSpline	20	0.0881	0.0903	−2.1610	2.6888 ± 0.5795	0.0881 ± 0.0201	0.01297 ± 0.00276	0.00381 ± 0.00071	0.01296 ± 0.00276	0.00488 ± 0.00089	1.154 × $10^{-8}$	6.479 × $10^{-8}$	1.944 × $10^{-7}$	Yes
OURS	20	0.0780	0.0817	−1.5873	2.3781 ± 0.7195	0.0780 ± 0.0246	0.00916 ± 0.00248	0.00313 ± 0.00058	0.00915 ± 0.00248	0.00381 ± 0.00072	–	–	–	Ref.
UPS	40	0.0248	0.0255	0.7474	0.7566 ± 0.1779	0.0248 ± 0.0062	0.00389 ± 0.00084	0.00100 ± 0.00017	0.00389 ± 0.00084	0.00129 ± 0.00021	1.071 × $10^{-30}$	8.024 × $10^{-18}$	4.814 × $10^{-17}$	Yes
UAS	40	0.0227	0.0232	0.7919	0.6924 ± 0.1373	0.0227 ± 0.0048	0.00487 ± 0.00141	0.00105 ± 0.00021	0.00487 ± 0.00141	0.00141 ± 0.00030	4.543 × $10^{-12}$	3.131 × $10^{-10}$	6.263 × $10^{-10}$	Yes
CBS	40	0.0337	0.0383	0.4331	1.0272 ± 0.5399	0.0337 ± 0.0181	0.00342 ± 0.00093	0.00099 ± 0.00019	0.00342 ± 0.00093	0.00124 ± 0.00025	9.709 × $10^{-16}$	1.378 × $10^{-14}$	4.133 × $10^{-14}$	Yes
DAAS	40	0.0550	0.0580	−0.3051	1.6767 ± 0.5449	0.0550 ± 0.0187	0.00569 ± 0.00120	0.00137 ± 0.00022	0.00569 ± 0.00121	0.00188 ± 0.00031	4.183 × $10^{-43}$	3.897 × $10^{-18}$	3.117 × $10^{-17}$	Yes
MCPD	40	0.0275	0.0293	0.6682	0.8401 ± 0.2983	0.0275 ± 0.0100	0.00319 ± 0.00060	0.00099 ± 0.00018	0.00319 ± 0.00060	0.00124 ± 0.00023	1.502 × $10^{-18}$	5.051 × $10^{-15}$	2.021 × $10^{-14}$	Yes
MECH	40	0.3957	0.5812	−129.8686	12.1035 ± 13.0952	0.3957 ± 0.4279	0.04326 ± 0.02619	0.01223 ± 0.01152	0.04326 ± 0.02619	0.01726 ± 0.01414	5.549 × $10^{-14}$	6.312 × $10^{-18}$	4.418 × $10^{-17}$	Yes
SEE	40	0.0250	0.0257	0.7436	0.7616 ± 0.1824	0.0250 ± 0.0063	0.00390 ± 0.00085	0.00100 ± 0.00017	0.00390 ± 0.00085	0.00129 ± 0.00022	1.835 × $10^{-27}$	8.024 × $10^{-18}$	4.814 × $10^{-17}$	Yes
CC-BSpline	40	0.0242	0.0251	0.7550	0.7387 ± 0.2010	0.0242 ± 0.0068	0.00493 ± 0.00137	0.00113 ± 0.00020	0.00493 ± 0.00137	0.00148 ± 0.00028	2.986 × $10^{-9}$	9.744 × $10^{-9}$	9.744e × $10^{-9}$	Yes
OURS	40	0.0208	0.0216	0.8190	0.6352 ± 0.1702	0.0208 ± 0.0058	0.00234 ± 0.00059	0.00083 ± 0.00015	0.00233 ± 0.00059	0.00098 ± 0.00018	–	–	–	Ref.
UPS	60	0.0112	0.0116	0.9482	0.3421 ± 0.0821	0.0112 ± 0.0028	0.00190 ± 0.00038	0.00045 ± 0.00008	0.00190 ± 0.00038	0.00058 ± 0.00010	1.179 × $10^{-31}$	7.117 × $10^{-18}$	4.270 × $10^{-17}$	Yes
UAS	60	0.0101	0.0103	0.9592	0.3070 ± 0.0591	0.0101 ± 0.0021	0.00271 ± 0.00099	0.00048 ± 0.00010	0.00271 ± 0.00099	0.00067 ± 0.00015	1.115 × $10^{-8}$	1.043 × $10^{-7}$	2.086 × $10^{-7}$	Yes
CBS	60	0.0153	0.0171	0.8861	0.4666 ± 0.2300	0.0153 ± 0.0077	0.00181 ± 0.00046	0.00048 ± 0.00009	0.00180 ± 0.00046	0.00062 ± 0.00012	1.073 × $10^{-17}$	7.398 × $10^{-15}$	2.219 × $10^{-14}$	Yes
DAAS	60	0.0366	0.0389	0.4152	1.1161 ± 0.3873	0.0366 ± 0.0131	0.00453 ± 0.00110	0.00090 ± 0.00016	0.00453 ± 0.00110	0.00133 ± 0.00025	3.915 × $10^{-44}$	3.897 × $10^{-18}$	3.117 × $10^{-17}$	Yes
MCPD	60	0.0129	0.0135	0.9294	0.3921 ± 0.1229	0.0129 ± 0.0042	0.00147 ± 0.00026	0.00045 ± 0.00008	0.00146 ± 0.00026	0.00057 ± 0.00010	4.219 × $10^{-29}$	5.943 × $10^{-18}$	4.160 × $10^{-17}$	Yes
MECH	60	0.2876	0.4968	−94.6061	8.8047 ± 12.4716	0.2876 ± 0.4071	0.03492 ± 0.02793	0.00940 ± 0.01170	0.03492 ± 0.02793	0.01343 ± 0.01455	6.852 × $10^{-10}$	8.519 × $10^{-18}$	4.270 × $10^{-17}$	Yes
SEE	60	0.0112	0.0116	0.9482	0.3421 ± 0.0821	0.0112 ± 0.0028	0.00190 ± 0.00038	0.00045 ± 0.00008	0.00190 ± 0.00038	0.00058 ± 0.00010	1.179 × $10^{-31}$	7.117 × $10^{-18}$	4.270 × $10^{-17}$	Yes
CC-BSpline	60	0.0116	0.0126	0.9385	0.3540 ± 0.1486	0.0116 ± 0.0049	0.00288 ± 0.00093	0.00062 ± 0.00008	0.00288 ± 0.00093	0.00080 ± 0.00013	8.081 × $10^{-6}$	6.346 × $10^{-6}$	6.346 × $10^{-6}$	Yes
OURS	60	0.0094	0.0098	0.9630	0.2870 ± 0.0775	0.0094 ± 0.0027	0.00109 ± 0.00026	0.00037 ± 0.00007	0.00107 ± 0.00027	0.00044 ± 0.00008	–	–	–	Ref.
UPS	80	0.0063	0.0065	0.9834	0.1937 ± 0.0467	0.0063 ± 0.0016	0.00114 ± 0.00022	0.00026 ± 0.00004	0.00113 ± 0.00023	0.00033 ± 0.00006	2.719 × $10^{-33}$	4.266 × $10^{-18}$	3.117 × $10^{-17}$	Yes
UAS	80	0.0056	0.0057	0.9872	0.1723 ± 0.0317	0.0056 ± 0.0011	0.00173 ± 0.00067	0.00028 ± 0.00006	0.00172 ± 0.00067	0.00039 ± 0.00009	4.053 × $10^{-10}$	4.856 × $10^{-9}$	9.712 × $10^{-9}$	Yes
CBS	80	0.0089	0.0102	0.9593	0.2698 ± 0.1535	0.0089 ± 0.0052	0.00117 ± 0.00029	0.00029 ± 0.00005	0.00116 ± 0.00029	0.00037 ± 0.00007	1.617 × $10^{-14}$	8.473 × $10^{-15}$	2.542 × $10^{-14}$	Yes
DAAS	80	0.0258	0.0278	0.7006	0.7856 ± 0.3087	0.0258 ± 0.0105	0.00373 ± 0.00104	0.00065 ± 0.00013	0.00373 ± 0.00104	0.00101 ± 0.00022	2.437 × $10^{-39}$	3.897 × $10^{-18}$	3.117 × $10^{-17}$	Yes
MCPD	80	0.0075	0.0080	0.9753	0.2285 ± 0.0814	0.0075 ± 0.0028	0.00085 ± 0.00015	0.00026 ± 0.00005	0.00083 ± 0.00015	0.00032 ± 0.00006	3.794 × $10^{-26}$	4.532 × $10^{-18}$	3.117 × $10^{-17}$	Yes
MECH	80	0.2042	0.4347	−72.1886	6.2315 ± 11.7878	0.2042 ± 0.3856	0.02512 ± 0.02808	0.00666 ± 0.01168	0.02512 ± 0.02808	0.00952 ± 0.01454	1.264 × $10^{-6}$	4.139 × $10^{-18}$	3.117 × $10^{-17}$	Yes
SEE	80	0.0063	0.0065	0.9834	0.1937 ± 0.0467	0.0063 ± 0.0016	0.00114 ± 0.00022	0.00026 ± 0.00004	0.00113 ± 0.00023	0.00033 ± 0.00006	2.719 × $10^{-33}$	4.266 × $10^{-18}$	3.117 × $10^{-17}$	Yes
CC-BSpline	80	0.0075	0.0084	0.9724	0.2290 ± 0.1175	0.0075 ± 0.0039	0.00188 ± 0.00057	0.00045 ± 0.00005	0.00187 ± 0.00057	0.00056 ± 0.00007	3.235 × $10^{-8}$	3.233 × $10^{-7}$	3.233 × $10^{-7}$	Yes
OURS	80	0.0053	0.0055	0.9884	0.1610 ± 0.0414	0.0053 ± 0.0014	0.00064 ± 0.00014	0.00021 ± 0.00004	0.00061 ± 0.00015	0.00025 ± 0.00005	–	–	–	Ref.
UPS	100	0.0041	0.0042	0.9932	0.1242 ± 0.0301	0.0041 ± 0.0010	0.00078 ± 0.00016	0.00016 ± 0.00003	0.00076 ± 0.00017	0.00021 ± 0.00004	6.805 × $10^{-35}$	4.671 × $10^{-18}$	3.117 × $10^{-17}$	Yes
UAS	100	0.0036	0.0037	0.9948	0.1101 ± 0.0205	0.0036 ± 0.0007	0.00120 ± 0.00046	0.00018 ± 0.00004	0.00119 ± 0.00047	0.00025 ± 0.00006	3.075 × $10^{-9}$	3.419 × $10^{-9}$	6.838 × $10^{-9}$	Yes
CBS	100	0.0057	0.0064	0.9841	0.1739 ± 0.0867	0.0057 ± 0.0029	0.00084 ± 0.00021	0.00020 ± 0.00004	0.00082 ± 0.00021	0.00026 ± 0.00005	1.198 × $10^{-17}$	8.473 × $10^{-15}$	2.542 × $10^{-14}$	Yes
DAAS	100	0.0224	0.0240	0.7761	0.6826 ± 0.2604	0.0224 ± 0.0088	0.00368 ± 0.00111	0.00055 ± 0.00012	0.00368 ± 0.00111	0.00092 ± 0.00022	1.134 × $10^{-41}$	3.897 × $10^{-18}$	3.117 × $10^{-17}$	Yes
MCPD	100	0.0047	0.0050	0.9903	0.1446 ± 0.0468	0.0047 ± 0.0016	0.00057 ± 0.00009	0.00017 ± 0.00003	0.00053 ± 0.00010	0.00021 ± 0.00004	3.314 × $10^{-30}$	4.016 × $10^{-18}$	3.117 × $10^{-17}$	Yes
MECH	100	0.1364	0.2771	−28.7434	4.1529 ± 7.3515	0.1364 ± 0.2424	0.01733 ± 0.01823	0.00420 ± 0.00534	0.01733 ± 0.01824	0.00621 ± 0.00749	3.124 × $10^{-7}$	4.266 × $10^{-18}$	3.117 × $10^{-17}$	Yes
SEE	100	0.0041	0.0042	0.9932	0.1242 ± 0.0301	0.0041 ± 0.0010	0.00078 ± 0.00016	0.00016 ± 0.00003	0.00076 ± 0.00017	0.00021 ± 0.00004	6.805 × $10^{-35}$	4.671 × $10^{-18}$	3.117 × $10^{-17}$	Yes
CC-BSpline	100	0.0051	0.0062	0.9853	0.1561 ± 0.1048	0.0051 ± 0.0035	0.00147 ± 0.00039	0.00039 ± 0.00003	0.00146 ± 0.00039	0.00048 ± 0.00004	1.907 × $10^{-6}$	1.222 × $10^{-5}$	1.222 × $10^{-5}$	Yes
OURS	100	0.0034	0.0035	0.9952	0.1035 ± 0.0267	0.0034 ± 0.0009	0.00045 ± 0.00008	0.00014 ± 0.00002	0.00040 ± 0.00010	0.00016 ± 0.00003	–	–	–	Ref.

All uncertainty terms are mean ± standard deviation. Paired significance tests compare absolute area error against OURS at the same *N*; Holm correction is applied across the method-wise Wilcoxon tests.

**Table 5:** Per-budget results of the droplet dataset.

Method	*N*	MAE	RMSE	$R^{2}$	Rel. Err. (%)	Abs. Err.	Haus.	NMCD	NMCD_max_	Fid. RMS	$p_{t}$	$p_{W}$	$p_{\mathrm{Holm}}$	Sig.
UPS	20	552.4316	627.6179	0.9984	1.7360 ± 0.2344	552.4316 ± 300.1131	0.00653 ± 0.00212	0.00183 ± 0.00044	0.00652 ± 0.00212	0.00238 ± 0.00059	8.312 × $10^{-5}$	2.634 × $10^{-6}$	7.902 × $10^{-6}$	Yes
UAS	20	546.4246	618.6407	0.9984	1.7240 ± 0.2154	546.4246 ± 292.2521	0.00660 ± 0.00218	0.00184 ± 0.00044	0.00660 ± 0.00218	0.00238 ± 0.00060	4.511 × $10^{-4}$	6.738 × $10^{-5}$	1.348 × $10^{-4}$	Yes
CBS	20	663.0266	787.6616	0.9974	1.9906 ± 0.5155	663.0266 ± 428.4228	0.00593 ± 0.00211	0.00186 ± 0.00046	0.00593 ± 0.00211	0.00236 ± 0.00064	2.714 × $10^{-9}$	1.295 × $10^{-10}$	7.768 × $10^{-10}$	Yes
DAAS	20	1217.5872	1505.3223	0.9905	3.6027 ± 1.3569	1217.5872 ± 891.8227	0.01340 ± 0.00635	0.00302 ± 0.00115	0.01340 ± 0.00635	0.00450 ± 0.00202	1.450 × $10^{-12}$	1.120 × $10^{-12}$	8.958 × $10^{-12}$	Yes
MCPD	20	513.1392	582.8363	0.9986	1.6704 ± 0.3317	513.1392 ± 278.4666	0.00565 ± 0.00134	0.00174 ± 0.00033	0.00565 ± 0.00134	0.00225 ± 0.00045	0.343	0.23	0.23	No
MECH	20	2556.0518	6641.1382	0.8155	6.4454 ± 13.3995	2556.0518 ± 6175.8058	0.01796 ± 0.03445	0.00593 ± 0.01415	0.01796 ± 0.03445	0.00786 ± 0.01807	0.0073	9.785 × $10^{-9}$	4.892 × $10^{-8}$	Yes
SEE	20	802.4002	947.0560	0.9962	2.4739 ± 0.7484	802.4002 ± 506.8563	0.00863 ± 0.00323	0.00233 ± 0.00068	0.00863 ± 0.00323	0.00312 ± 0.00096	1.682 × $10^{-11}$	7.253 × $10^{-12}$	5.077 × $10^{-11}$	Yes
CC-BSpline	20	568.2760	646.2045	0.9983	1.7858 ± 0.2491	568.2760 ± 309.9622	0.00668 ± 0.00215	0.00193 ± 0.00044	0.00667 ± 0.00215	0.00245 ± 0.00060	5.205 × $10^{-6}$	2.237 × $10^{-7}$	8.949 × $10^{-7}$	Yes
OURS	20	495.9792	568.4817	0.9986	1.5486 ± 0.2342	495.9792 ± 279.9026	0.00496 ± 0.00168	0.00165 ± 0.00042	0.00496 ± 0.00168	0.00202 ± 0.00053	–	–	–	Ref.
UPS	40	140.1505	158.2488	0.9999	0.4383 ± 0.0313	140.1505 ± 74.0427	0.00239 ± 0.00079	0.00055 ± 0.00014	0.00238 ± 0.00079	0.00073 ± 0.00019	8.905 × $10^{-19}$	1.681 × $10^{-12}$	1.176 × $10^{-11}$	Yes
UAS	40	139.0067	156.4067	0.9999	0.4371 ± 0.0230	139.0067 ± 72.2362	0.00265 ± 0.00124	0.00056 ± 0.00015	0.00265 ± 0.00124	0.00076 ± 0.00023	1.366 × $10^{-17}$	2.405 × $10^{-12}$	1.202 × $10^{-11}$	Yes
CBS	40	168.2657	200.5184	0.9998	0.5096 ± 0.1446	168.2657 ± 109.8840	0.00199 ± 0.00073	0.00055 ± 0.00015	0.00198 ± 0.00073	0.00071 ± 0.00022	1.974 × $10^{-10}$	1.452 × $10^{-11}$	5.808 × $10^{-11}$	Yes
DAAS	40	268.2464	303.4472	0.9996	0.8872 ± 0.2082	268.2464 ± 142.9301	0.00441 ± 0.00112	0.00083 ± 0.00015	0.00441 ± 0.00112	0.00128 ± 0.00026	1.224 × $10^{-19}$	1.172 × $10^{-12}$	9.372 × $10^{-12}$	Yes
MCPD	40	141.0437	163.7900	0.9999	0.4329 ± 0.0862	141.0437 ± 83.8981	0.00174 ± 0.00053	0.00051 ± 0.00014	0.00174 ± 0.00053	0.00065 ± 0.00018	3.163 × $10^{-8}$	7.838 × $10^{-9}$	1.568 × $10^{-8}$	Yes
MECH	40	257.4636	1085.5483	0.9951	0.6913 ± 2.3679	257.4636 ± 1062.5338	0.00307 ± 0.00714	0.00075 ± 0.00188	0.00306 ± 0.00715	0.00101 ± 0.00269	0.273	5.515 × $10^{-4}$	5.515 × $10^{-4}$	Yes
SEE	40	140.1505	158.2488	0.9999	0.4383 ± 0.0313	140.1505 ± 74.0427	0.00239 ± 0.00079	0.00055 ± 0.00014	0.00238 ± 0.00079	0.00073 ± 0.00019	8.905 × $10^{-19}$	1.681 × $10^{-12}$	1.176 × $10^{-11}$	Yes
CC-BSpline	40	154.0651	177.9211	0.9999	0.4872 ± 0.1042	154.0651 ± 89.6653	0.00287 ± 0.00119	0.00072 ± 0.00014	0.00286 ± 0.00119	0.00091 ± 0.00020	2.209 × $10^{-9}$	7.606 × $10^{-10}$	2.282 × $10^{-9}$	Yes
OURS	40	114.9749	130.8414	0.9999	0.3571 ± 0.0413	114.9749 ± 62.9233	0.00130 ± 0.00042	0.00044 ± 0.00012	0.00129 ± 0.00043	0.00053 ± 0.00014	–	–	–	Ref.
UPS	60	62.2980	70.2917	1.0000	0.1950 ± 0.0136	62.2980 ± 32.8015	0.00121 ± 0.00042	0.00025 ± 0.00007	0.00120 ± 0.00042	0.00034 ± 0.00009	1.288 × $10^{-20}$	1.120 × $10^{-12}$	8.958 × $10^{-12}$	Yes
UAS	60	61.7272	69.3382	1.0000	0.1944 ± 0.0086	61.7272 ± 31.8222	0.00143 ± 0.00068	0.00026 ± 0.00007	0.00142 ± 0.00068	0.00036 ± 0.00011	5.552 × $10^{-20}$	1.120 × $10^{-12}$	8.958 × $10^{-12}$	Yes
CBS	60	76.7409	91.5431	1.0000	0.2342 ± 0.0725	76.7409 ± 50.2863	0.00114 ± 0.00043	0.00028 ± 0.00008	0.00112 ± 0.00043	0.00036 ± 0.00011	1.098 × $10^{-10}$	2.758 × $10^{-11}$	8.275 × $10^{-11}$	Yes
DAAS	60	168.5404	187.9578	0.9999	0.5674 ± 0.1288	168.5404 ± 83.8280	0.00325 ± 0.00080	0.00053 ± 0.00009	0.00324 ± 0.00080	0.00087 ± 0.00017	9.425 × $10^{-24}$	1.120 × $10^{-12}$	8.958 × $10^{-12}$	Yes
MCPD	60	62.6195	72.5161	1.0000	0.1903 ± 0.0326	62.6195 ± 36.8461	0.00082 ± 0.00022	0.00024 ± 0.00006	0.00081 ± 0.00023	0.00030 ± 0.00008	3.626 × $10^{-12}$	9.839 × $10^{-12}$	3.935 × $10^{-11}$	Yes
MECH	60	59.2573	68.9843	1.0000	0.1839 ± 0.0390	59.2573 ± 35.5851	0.00103 ± 0.00068	0.00024 ± 0.00007	0.00101 ± 0.00069	0.00031 ± 0.00011	1.625 × $10^{-5}$	6.041 × $10^{-9}$	1.208 × $10^{-8}$	Yes
SEE	60	62.2980	70.2917	1.0000	0.1950 ± 0.0136	62.2980 ± 32.8015	0.00121 ± 0.00042	0.00025 ± 0.00007	0.00120 ± 0.00042	0.00034 ± 0.00009	1.288 × $10^{-20}$	1.120 × $10^{-12}$	8.958 × $10^{-12}$	Yes
CC-BSpline	60	72.4533	88.7192	1.0000	0.2336 ± 0.0970	72.4533 ± 51.5881	0.00180 ± 0.00062	0.00048 ± 0.00006	0.00179 ± 0.00062	0.00059 ± 0.00008	9.555 × $10^{-6}$	3.463 × $10^{-6}$	3.463 × $10^{-6}$	Yes
OURS	60	50.4658	57.5504	1.0000	0.1564 ± 0.0187	50.4658 ± 27.8719	0.00061 ± 0.00019	0.00020 ± 0.00005	0.00058 ± 0.00020	0.00024 ± 0.00006	–	–	–	Ref.
UPS	80	35.0670	39.5854	1.0000	0.1097 ± 0.0080	35.0670 ± 18.5046	0.00076 ± 0.00026	0.00015 ± 0.00004	0.00074 ± 0.00026	0.00020 ± 0.00005	1.104 × $10^{-20}$	1.120 × $10^{-12}$	8.958 × $10^{-12}$	Yes
UAS	80	34.6951	38.9603	1.0000	0.1093 ± 0.0048	34.6951 ± 17.8583	0.00087 ± 0.00042	0.00015 ± 0.00004	0.00086 ± 0.00043	0.00021 ± 0.00006	3.703 × $10^{-21}$	1.120 × $10^{-12}$	8.958 × $10^{-12}$	Yes
CBS	80	45.2794	54.4200	1.0000	0.1370 ± 0.0431	45.2794 ± 30.4157	0.00075 ± 0.00030	0.00017 ± 0.00005	0.00072 ± 0.00031	0.00022 ± 0.00007	2.194 × $10^{-10}$	2.533 × $10^{-11}$	7.600 × $10^{-11}$	Yes
DAAS	80	122.7757	140.2286	0.9999	0.4067 ± 0.1153	122.7757 ± 68.2624	0.00263 ± 0.00092	0.00038 ± 0.00008	0.00262 ± 0.00092	0.00065 ± 0.00018	4.978 × $10^{-21}$	1.120 × $10^{-12}$	8.958 × $10^{-12}$	Yes
MCPD	80	34.8283	40.3163	1.0000	0.1064 ± 0.0180	34.8283 ± 20.4608	0.00051 ± 0.00011	0.00014 ± 0.00004	0.00046 ± 0.00013	0.00017 ± 0.00005	5.174 × $10^{-13}$	3.751 × $10^{-12}$	1.500 × $10^{-11}$	Yes
MECH	80	32.1081	37.3230	1.0000	0.0989 ± 0.0210	32.1081 ± 19.1719	0.00053 ± 0.00019	0.00013 ± 0.00003	0.00049 ± 0.00020	0.00016 ± 0.00004	1.545 × $10^{-5}$	8.683 × $10^{-6}$	8.683 × $10^{-6}$	Yes
SEE	80	35.0670	39.5854	1.0000	0.1097 ± 0.0080	35.0670 ± 18.5046	0.00076 ± 0.00026	0.00015 ± 0.00004	0.00074 ± 0.00026	0.00020 ± 0.00005	1.104 × $10^{-20}$	1.120 × $10^{-12}$	8.958 × $10^{-12}$	Yes
CC-BSpline	80	50.8416	64.0371	1.0000	0.1599 ± 0.0786	50.8416 ± 39.2282	0.00140 ± 0.00035	0.00041 ± 0.00004	0.00138 ± 0.00036	0.00050 ± 0.00005	2.073 × $10^{-7}$	5.448 × $10^{-7}$	1.090 × $10^{-6}$	Yes
OURS	80	28.4084	32.4161	1.0000	0.0878 ± 0.0105	28.4084 ± 15.7309	0.00040 ± 0.00009	0.00012 ± 0.00003	0.00033 ± 0.00011	0.00014 ± 0.00004	–	–	–	Ref.
UPS	100	22.4803	25.3863	1.0000	0.0703 ± 0.0052	22.4803 ± 11.8831	0.00054 ± 0.00018	0.00009 ± 0.00003	0.00050 ± 0.00019	0.00013 ± 0.00004	8.243 × $10^{-22}$	1.120 × $10^{-12}$	8.958 × $10^{-12}$	Yes
UAS	100	22.1967	24.9210	1.0000	0.0700 ± 0.0029	22.1967 ± 11.4152	0.00066 ± 0.00037	0.00010 ± 0.00003	0.00063 ± 0.00038	0.00014 ± 0.00004	7.230 × $10^{-22}$	1.120 × $10^{-12}$	8.958 × $10^{-12}$	Yes
CBS	100	29.1510	35.6933	1.0000	0.0883 ± 0.0340	29.1510 ± 20.7523	0.00058 ± 0.00021	0.00011 ± 0.00003	0.00054 ± 0.00023	0.00015 ± 0.00005	2.567 × $10^{-9}$	3.705 × $10^{-9}$	1.112 × $10^{-8}$	Yes
DAAS	100	97.7893	112.2486	0.9999	0.3329 ± 0.1152	97.7893 ± 55.5249	0.00245 ± 0.00091	0.00031 ± 0.00007	0.00244 ± 0.00091	0.00057 ± 0.00017	2.272 × $10^{-20}$	1.172 × $10^{-12}$	8.958 × $10^{-12}$	Yes
MCPD	100	22.2343	25.5488	1.0000	0.0682 ± 0.0106	22.2343 ± 12.6798	0.00037 ± 0.00006	0.00009 ± 0.00002	0.00030 ± 0.00009	0.00011 ± 0.00003	5.297 × $10^{-15}$	5.338 × $10^{-12}$	2.135 × $10^{-11}$	Yes
MECH	100	19.9371	23.1618	1.0000	0.0614 ± 0.0126	19.9371 ± 11.8780	0.00040 ± 0.00028	0.00009 ± 0.00003	0.00033 ± 0.00029	0.00011 ± 0.00004	1.488 × $10^{-4}$	1.418 × $10^{-6}$	1.418 × $10^{-6}$	Yes
SEE	100	22.4803	25.3863	1.0000	0.0703 ± 0.0052	22.4803 ± 11.8831	0.00054 ± 0.00018	0.00009 ± 0.00003	0.00050 ± 0.00019	0.00013 ± 0.00004	8.243 × $10^{-22}$	1.120 × $10^{-12}$	8.958 × $10^{-12}$	Yes
CC-BSpline	100	36.8096	46.3086	1.0000	0.1152 ± 0.0640	36.8096 ± 28.3108	0.00124 ± 0.00029	0.00039 ± 0.00003	0.00123 ± 0.00029	0.00047 ± 0.00004	7.441 × $10^{-9}$	1.464 × $10^{-8}$	2.928 × $10^{-8}$	Yes
OURS	100	17.8017	20.2932	1.0000	0.0551 ± 0.0063	17.8017 ± 9.8160	0.00031 ± 0.00004	0.00008 ± 0.00002	0.00021 ± 0.00007	0.00009 ± 0.00002	–	–	–	Ref.

All uncertainty terms are mean ± standard deviation. Paired significance tests compare absolute area error against OURS at the same *N*; Holm correction is applied across the method-wise Wilcoxon tests.

**Table 6:** Sampling-budget estimation under target area-error tolerance constraints.

Target Relative Error Tolerance (%)	Estimated *N* Mean	Estimated *N* Std	Achieved Relative Error (%)	Std. Relative Error (%)	Satisfaction Rate
0.1	119.32	19.29	0.073276	0.011957	0.97
0.2	84.54	13.64	0.145714	0.023295	0.97
0.3	69.11	11.173	0.216574	0.035757	0.99
0.4	59.94	9.636	0.286585	0.045626	0.99
0.5	53.71	8.645	0.361512	0.062662	0.97
0.6	49.01	7.861	0.434118	0.073592	0.97
0.7	45.39	7.250	0.496185	0.085220	0.98
0.8	42.55	6.866	0.568375	0.096161	0.99
0.9	40.13	6.449	0.636818	0.112578	0.98
1.0	38.09	6.099	0.698039	0.114763	1.00

## Data Availability

The original contributions presented in this study are included in the article. Further inquiries can be directed to the corresponding author.
